# Antioxidant Metabolism Underlies Different Metabolic Strategies for Primary Root Growth Maintenance under Water Stress in Cotton and Maize

**DOI:** 10.3390/antiox11050820

**Published:** 2022-04-22

**Authors:** Jian Kang, Priyamvada Voothuluru, Elizabeth Hoyos-Miernyk, Danny Alexander, Melvin J. Oliver, Robert E. Sharp

**Affiliations:** 1 Division of Plant Science and Technology, University of Missouri, Columbia, MO 65211, USA; jkf64@umsystem.edu (J.K.); hoyosm@missouri.edu (E.H.-M.); olivermj@missouri.edu (M.J.O.); 2 Interdisciplinary Plant Group, University of Missouri, Columbia, MO 65211, USA; 3 Center for Renewable Carbon, University of Tennessee Institute of Agriculture, Knoxville, TN 37996, USA; pvoothul@utk.edu; 4 Metabolon Inc., Durham, NC 27713, USA; dalexander@metabolon.com

**Keywords:** antioxidative metabolism, glutathione, *Gossypium hirsutum*, metabolomics, roots, sulfur metabolism, water stress, *Zea mays*

## Abstract

The divergence of metabolic responses to water stress in the elongation zone of cotton and maize primary roots was investigated by establishing water-deficit conditions that generated steady root elongation at equivalent tissue water potentials. In water-stressed cotton roots, cell elongation was maintained in the apical 3 mm but was progressively inhibited with further displacement from the apex. These responses are similar to previous findings in maize, providing the foundation for comparisons of metabolic responses in regions of growth maintenance and inhibition between the species. Metabolomics analyses showed region-specific and species-specific changes in metabolite abundance in response to water stress, revealing both conserved responses including osmolyte accumulation, and key differences in antioxidative and sulfur metabolism. Quantitative assessment showed contrasting glutathione responses in the root elongation zone between the species, with glutathione levels declining in cotton as stress duration progressed, whereas in maize, glutathione levels remained elevated. Despite the lesser glutathione response in cotton, hydrogen peroxide levels were low in water-stressed cotton compared with maize roots and were associated with higher catalase, ascorbate peroxidase, and superoxide dismutase activities in cotton. The results indicate alternative metabolic strategies underlying the responses of primary root growth to water stress between cotton and maize.

## 1. Introduction

Plants respond to water stress by establishing cellular conditions to defend against the negative effects of the stress and, generally, by suppressing growth and development [1]. However, root growth is usually less inhibited than shoot growth, which helps to maintain access to soil water at depth and thus to survive drought conditions [2]. In the maize (*Zea mays* L.) primary root, this response is characterized by the maintenance of cell expansion in the apical region of the elongation zone, which also contains the meristem, whereas growth is inhibited as cells are displaced further from the apex [3]. The consequence of these responses is that roots continue to grow, albeit at a reduced rate, at water potentials that completely inhibit shoot growth. Although it is documented in several crop species that primary roots can continue to grow under severe water-deficit conditions [3,4,5,6], it is not known if the mechanisms by which this is achieved are similar to those in maize, which has been studied extensively [7].

Cotton (*Gossypium hirsutum* L.) is an important economic crop that is considered to be relatively drought tolerant [8]. Nevertheless, cotton production faces a growing threat of increased yield losses resulting from water limitation [9], and there is major interest in understanding and improving water stress tolerance in this crop [10]. Cotton is a dicotyledonous perennial plant that is grown commercially as an annual, and production is highly dependent on early seedling growth and development [11]. Cotton has relatively poor seedling vigor, however, and is vulnerable to early season stress events [12], leading to poor stand establishment.

Root morphology and growth characteristics are important for cotton adaptation to water-limited conditions [13,14]. Growth of the primary or “tap” root, as the foundation of the root system, is critical to establishment, and seedling root growth characteristics have been used successfully to screen for drought-tolerant germplasm [15,16]. With early-season water limitation, the primary root needs to penetrate through dry soil, and it has been reported that primary root elongation was maintained [17] or increased [18] under soil water-deficit conditions. It was also shown that primary root growth continued, albeit at reduced rates compared to well-watered controls, at much lower soil water potentials than those that completely inhibited shoot growth [4]. For cotton, however, information is lacking on the physiological and molecular mechanisms that determine the responses of primary root growth to water deficits.

Here, we investigated the divergence of metabolic responses between cotton and maize primary roots growing under water stress. A key challenge for comparative studies of the effects of water deficits is achieving reproducible responses at similar stress levels and developmental stages. This strategy is necessary to avoid differences in growth and metabolic responses as stress severity increases during soil drying. We established water-deficit conditions that generated steady primary root elongation at equivalent root tissue water potentials in cotton and maize seedlings. This approach allowed direct comparisons of spatial patterns of cell elongation and associated metabolic responses within the root elongation zones. Comparative metabolomics was used to investigate and contrast responses to water stress in the two species. Metabolomic analysis provides a powerful approach to comprehensively assess the regulation of plant metabolism and has provided novel insights into mechanisms of acclimation to water stress in other crops [19,20]. The results reveal both conserved aspects of the water deficit response as well as key differences in antioxidative and sulfur metabolism that indicate alternative metabolic strategies for primary root growth under water stress between cotton and maize.

## 2. Materials and Methods

### 2.1. Plant Materials and Growth Conditions

Cotton (cv. AU90810) and maize (cv. FR697) were grown using a vermiculite culture system [3], with modifications for cotton as detailed below. Seedlings with primary roots of 5–15 mm in length were transplanted against the sides of Plexiglas boxes containing vermiculite (no. 2A, Therm-O-Rock East Inc., New Eagle, PA, USA) at water potentials of −0.02 MPa (well-watered), −1.0 MPa (cotton) or −1.6 MPa (cotton and maize), which were obtained by mixing with varying amounts of 1 mM CaSO_4_ and measured by isopiestic thermocouple psychrometry [21]. Seedlings were grown at 29 °C and near-saturation humidity in the dark to minimize further drying of the media during the experiments. Primary root elongation was monitored by periodically marking the position of the root apices. Transplanting, growth measurements, and harvesting were performed using a green ‘safe’ light [22].

For cotton, preliminary experiments showed that primary root elongation progressively decreased after transplanting to low water potentials, particularly under severe stress. To test whether non-steady elongation resulted from inadequate hydraulic contact with the vermiculite [23], the particle size was sieved to 3 mm or less, which resulted in increased and stable root elongation after 24 h (Figure S1) and, therefore, was used for subsequent experiments. In maize, sieved vermiculite did not alter the root elongation response to low water potentials; therefore, non-sieved media was used as in previous studies.

### 2.2. Root Elongation Zone Water Potentials

The water potential of the primary root elongation zone was measured by isopiestic thermocouple psychrometry. Based on kinematic analyses (described below), the whole elongation zone plus 3–4 mm of mature tissue was harvested. The mature tissue provided a water source during the measurements, without which expanding cells could have exhibited wall relaxation and turgor decrease, resulting in erroneously low water potentials [24]. Samples comprised 3–5 root tips, which were harvested at near-saturation humidity to avoid dehydration.

Root water potentials were measured in a cold room at 5 °C. With cotton, this procedure prevented extremely long (>8 h) psychrometric equilibration times that eventuated in erroneously high water potentials when measurements were made at the standard system temperature of 22 °C. These effects apparently resulted from the exudation of liquid at the root surface, which may have had a higher water potential such that the psychrometer measured an average of root and exudate water potentials (exudate water potential could not be independently measured because amounts were small). At 5 °C, exudation was prevented, presumably due to inhibition of metabolic activity. In maize, root tip water potential measurements were unaffected by system temperature.

### 2.3. Kinematic Analysis

Spatial distributions of displacement velocity (mm h^−1^) within the elongation zone were calculated from root elongation rates and cell length profiles [25]. For cotton, experiments were conducted at vermiculite water potentials of −0.02, −1.0, and −1.6 MPa. For each treatment, the apical 20 mm of four to five roots with elongation rates close to the mean of 20 seedlings were collected at 48 h. Determination of displacement velocity profiles from anatomical records requires steady growth; root elongation rates were steady in all treatments after 24 h. Root tips were sectioned longitudinally (Vibratome 3000 Plus, Leica Biosystems, Deer Park, IL, USA), stained with Calcofluor-white (Sigma-Aldrich, St. Louis, MO, USA) for 15 min to visualize the cell walls, and imaged by confocal microscopy (Leica TCS SP8, Leica Microsystems, Buffalo Grove, IL, USA) at 250 µm intervals from the root cap junction. For each root, four to six cell lengths were measured at each position using ImageJ software (NIH, Bethesda, MD, USA). Final cell lengths were determined as the average of eight positions after unchanging lengths were obtained. For maize, previous data for cv. FR697 at 48 h after transplanting to well-watered or water-stressed (−1.6 MPa) conditions in the same system were utilized [26].

The distribution of displacement velocity for each root was calculated from *V*_A_ *= L*_A_ *× V*_F_/*L*_F_, where *L*_A_ is the cell length at position A, *L*_F_ is the final cell length, *V*_A_ is the velocity at position A, and *V*_F_ is the final velocity (equal to the root elongation rate). Displacement velocities could not be derived in the meristematic region where cell lengths are determined by both elongation and division and were therefore calculated from the distal end of the meristem (approximated to where cells reached 2.5 times the shortest length; [27]). Logistic curves were fitted to describe profiles of displacement velocity (OriginLab Corp., Northampton, MA, USA). Rates of cell flux (the rate at which cells exit the elongation zone; cells h^−1^) were calculated by dividing root elongation rates by final cell lengths.

### 2.4. Metabolomics Analysis

Cotton and maize primary root tips were harvested 48 h after transplanting to vermiculite water potentials of −1.0 MPa (cotton) and −1.6 MPa (maize), which resulted in equivalent root tip water potentials (see Results for details of comparative root tip water potentials). Well-watered developmental (approximately the same length as the water-stressed roots) and temporal (same age as the water-stressed roots) controls were harvested at 24 h and 48 h. Root tips were divided into three regions as characterized by the kinematic analysis (see Results). Segments were pooled to generate a minimum of 50 mg dry weight (DW) per sample and frozen in liquid nitrogen. Four biological replicates were collected for each treatment.

Samples were lyophilized, ground in liquid nitrogen, weighed, and sent to Metabolon Inc. (Durham, NC, USA) for unbiased global metabolomics profiling. The platform consisted of ultra-high performance liquid chromatography-tandem mass spectroscopy (cotton and maize) and gas chromatography-mass spectrometry (maize), as described by Evans et al. [28]. Samples were extracted in methanol containing recovery standards and prepared for analysis using an automated MicroLab STAR system (Hamilton Company, Reno, NV, USA), as described by Yobi et al. [29].

Raw data were extracted, peak-annotated, and QC-processed by Metabolon. Compounds were identified by comparison to library entries of purified standards or recurrent unknowns, based on retention time/index (RI), mass to charge ratio (m/z), and chromatographic data. Chromatographic separation, followed by full-scan mass spectra, was performed for all detectable ions present in the samples (Supplementary Data S1). Consistency of peak annotation among samples was confirmed by proprietary visualization and interpretation software. Library matches for each compound were checked manually for each sample.

### 2.5. Glutathione, Hydrogen Peroxide and Antioxidant Enzyme Assays

Cotton and maize primary roots were harvested 12, 24, 36, and 48 h after transplanting to well-watered or water-stressed conditions. Whole elongation zones were collected, frozen in liquid nitrogen, and pooled to generate 50–150 mg fresh weight (FW) per sample. Three to six replicates, depending on the assay, were collected for each treatment.

Glutathione was measured enzymatically using the dithio-bis-2-nitrobenzoic acid-glutathione reductase (DTNB-GR) recycling method, as described by Noctor et al. [30] with some modifications. Samples were ground in liquid nitrogen, extracted with 1.5 mL of 1 M HClO_4_, and centrifuged at 14,000 rpm for 15–20 min at 4 °C. Supernatant was collected and 120 mM KH_2_PO_4_ (pH 5.6) was added at a ratio of supernatant: solution of 5:1. The pH was adjusted to 5.6 using 5 M K_2_CO_3_. Samples were centrifuged to remove insoluble KClO4 and separated into two aliquots. Total glutathione was measured by the reaction of reduced glutathione (GSH) in 50 μL supernatant with 60 μL 10 mM DTNB after 10 μL glutathione reductase (0.5 Unit) was used to reduce all oxidized glutathione (GSSG) to GSH in the presence of 5 μL 100 mM NADPH in 885 μL buffer containing 120 mM K_2_HPO_4_ and 6 mM EDTA (pH 7.5). To measure GSSG, free GSH was removed by adding 2.5 μL 2-vinyl pyridine to 100 μL supernatant prior to the reduction of remaining GSSG to GSH for measurement with DNTB. GSH was determined from the absorbance at 412 nm and GSSG was derived by subtraction.

Hydrogen peroxide (H_2_O_2_) was measured as described by Le et al. [31], using an Amplex Red Hydrogen Peroxide/Peroxidase Assay Kit (Invitrogen, Thermo Fisher Scientific, Inc., Waltham, MA, USA). Samples were ground in liquid nitrogen, extracted with 50 mM sodium phosphate buffer (pH 7.4) at a ratio of sample (mg FW): buffer (μL) of 3:5, and centrifuged at 14,000 rpm for 10–15 min at 4 °C. Twenty-five microliters of supernatant was mixed with 25 μL of reaction buffer containing 0.25 μL Amplex Red, 0.25 μL horseradish peroxidase, and 24.5 μL 50 mM sodium phosphate buffer. The mixture was incubated for 30 min at room temperature, and H_2_O_2_ was determined from the absorbance at 560 nm.

Samples for antioxidant enzyme assays were ground in liquid nitrogen, extracted with 1 mL 50 mM potassium phosphate buffer containing 0.1 mM EDTA and 1% polyvinylpolypyrrolidone, and centrifuged at 14,000 rpm for 25 min at 4 °C. The supernatant was purified using a Sephadex column (Global Life Sciences Solutions USA LLC, Marlborough, MA, USA) and 50 μL aliquots were assayed. Total protein was measured using the Bradford assay [32]. Catalase (CAT) activity was determined as described by Aebi [33] and calculated from the change in absorbance (ΔA240) and the extinction coefficient of 0.0436 mM^−1^ cm^−1^. Ascorbate peroxidase (APX) activity was measured as described by Amako et al. [34] and calculated from ΔA290 and the extinction coefficient of 2.8 mM^−1^ cm^−1^. Superoxide dismutase (SOD) activity was measured as described by Giannopolitis and Ries [35]; one unit of activity was defined as the amount that inhibits 50% of the reduction of 75 nmol NBT.

### 2.6. Statistical Analyses

For metabolomics, raw data were normalized for internal consistency by processing a constant DW per volume of extraction solvent for each sample. Data were scaled to the median value for each compound. If the value for a given metabolite was missing in any replicate, minimum detected values were imputed based on the assumption that missing values were below the limits of detection. Statistical calculations used natural log-transformed scaled imputed data. ANOVA contrasts and Welch’s two-sample *t*-tests identified metabolites that differed significantly between experimental groups (*p* < 0.05 or <0.10 levels). Two-way ANOVA identified metabolites exhibiting significant interaction and main effects for experimental parameters of treatment and region. For other measurements, ANOVA analyses and *t*-tests were carried out in Minitab (Minitab, LLC, State College, PA, USA) to compare across time and treatments.

## 3. Results

### 3.1. Cotton and Maize Primary Root Growth Responses to Equivalent Tissue Water Stress

**Figure 1:**
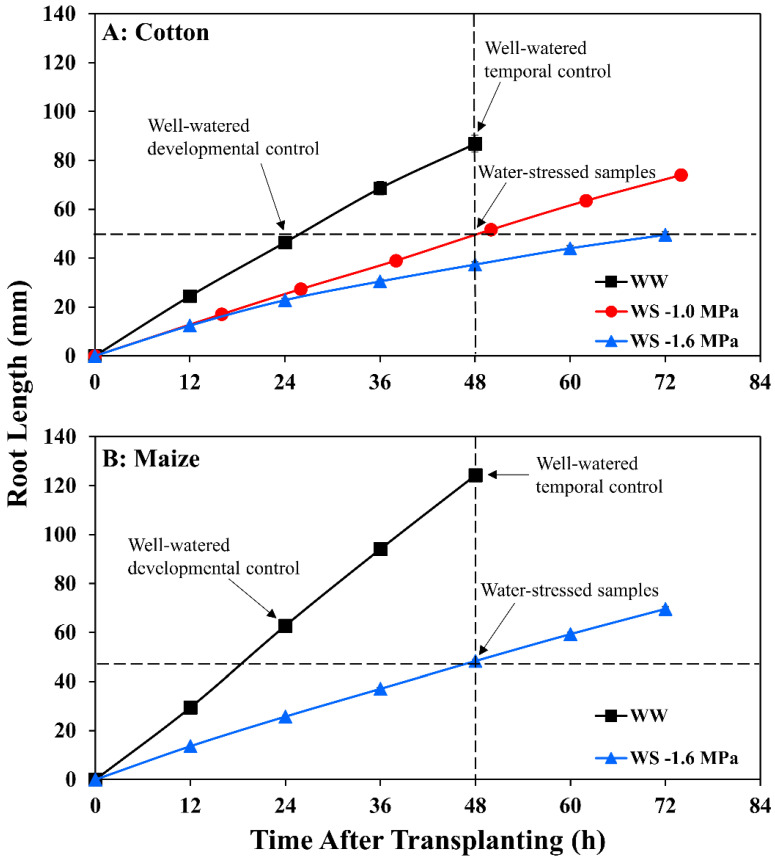
Time course of primary root length for (**A**) cotton and (**B**) maize seedlings after transplanting to well-watered (WW) or water-stressed (WS) conditions. In the water stress treatments, vermiculite water potentials were −1.0 MPa (cotton) and −1.6 MPa (cotton and maize). Data are the means ± SE of 32–48 seedlings. Primary root elongation rates were calculated from slopes of root length increase after 24 h when elongation was steady in all treatments. Arrows indicate times at which root samples were collected for metabolomic analyses in the water-stressed treatments and in the well-watered developmental control (approximately the same length as the water-stressed roots) and temporal control (same age as the water-stressed roots).

Maize seedlings of cv. FR697 were grown at a water potential of −1.6 MPa, a severe stress at which shoot growth was completely inhibited but root elongation continued at 39% of the well-watered rate (Figure 1B; Table S1). FR697 was chosen as it exhibited a relatively greater ability to maintain primary root elongation under water deficits [36] and was used previously to characterize patterns of relative elongation rate [26], transcript abundance [37], cell wall [38], and plasma membrane proteins [39], and apoplastic H_2_O_2_ [40] in well-watered and water-stressed roots.

For cotton, similarly, a water stress-tolerant line in terms of primary root elongation was selected from a comparison of nine genotypes provided by the Regional Breeders Testing Network (Cotton Inc., Cary, NC, USA). Of the nine genotypes, AU90810 exhibited the greatest ability to maintain root elongation under severe stress. Nevertheless, inhibition of elongation was greater than in maize, with only 26% of the well-watered rate occurring at a water potential of −1.6 MPa (Figure 1A; Table S1).

**Figure 2:**
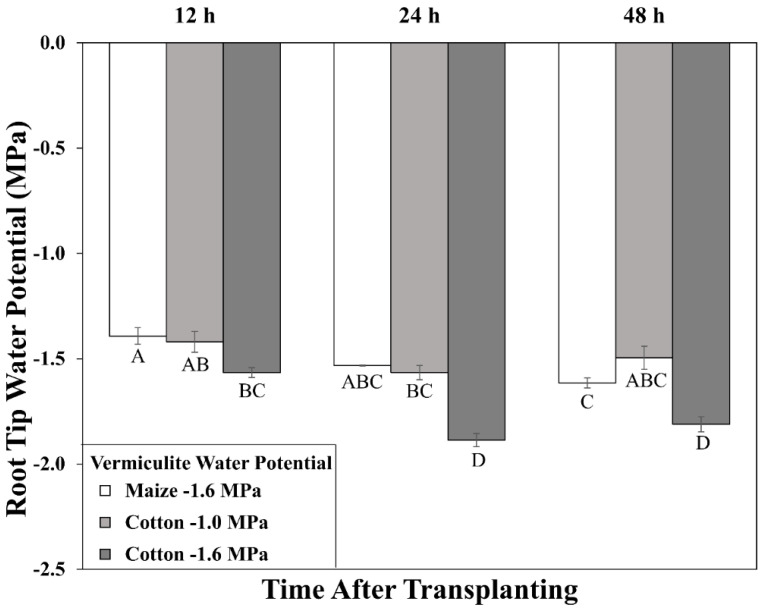
Time course of primary root tip (encompassing the elongation zone) water potential for cotton and maize seedlings after transplanting to water-stressed conditions. Vermiculite water potentials were −1.0 MPa (cotton) and −1.6 MPa (cotton and maize). Data are the means ± SE (*n* = 3–4). ANOVA analysis compares the data across treatments, time points, and species; different letters indicate significant differences (*p* < 0.01).

In maize, after transplanting to vermiculite at −1.6 MPa, the primary root tip water potential declined to a stable value of around −1.6 MPa by 24 h (Figure 2), in approximate equilibration with the medium and in agreement with previous results [41]. In association, root elongation was steady after 24 h under well-watered (2.6 mm h^−1^) and stressed (1.0 mm h^−1^) conditions (Figure 1B; Table S1).

In cotton growing at a vermiculite water potential of −1.6 MPa, however, the root tip water potential declined to a stable value of approximately −1.8 MPa, significantly lower than in maize (Figure 2). Thus, cotton roots were more severely stressed than maize roots when grown at the same media water potential, indicating greater hydraulic limitation to water uptake into the elongation zone (despite the use of sieved vermiculite; see Methods). Accordingly, growth conditions were established that achieved equivalent root tip water potentials in the two species. The results showed that transplanting cotton to vermiculite at −1.0 MPa resulted in steady root tip water potentials after 24 h that were not significantly different from those in maize growing at −1.6 MPa (Figure 2). Cotton root elongation rate at −1.0 MPa (1.04 mm h^−1^) was also similar to maize roots growing at −1.6 MPa (1.00 mm h^−1^; Figure 1; Table S1).

### 3.2. Kinematic Analysis of Cell Elongation Profiles and Cell Flux

**Figure 3:**
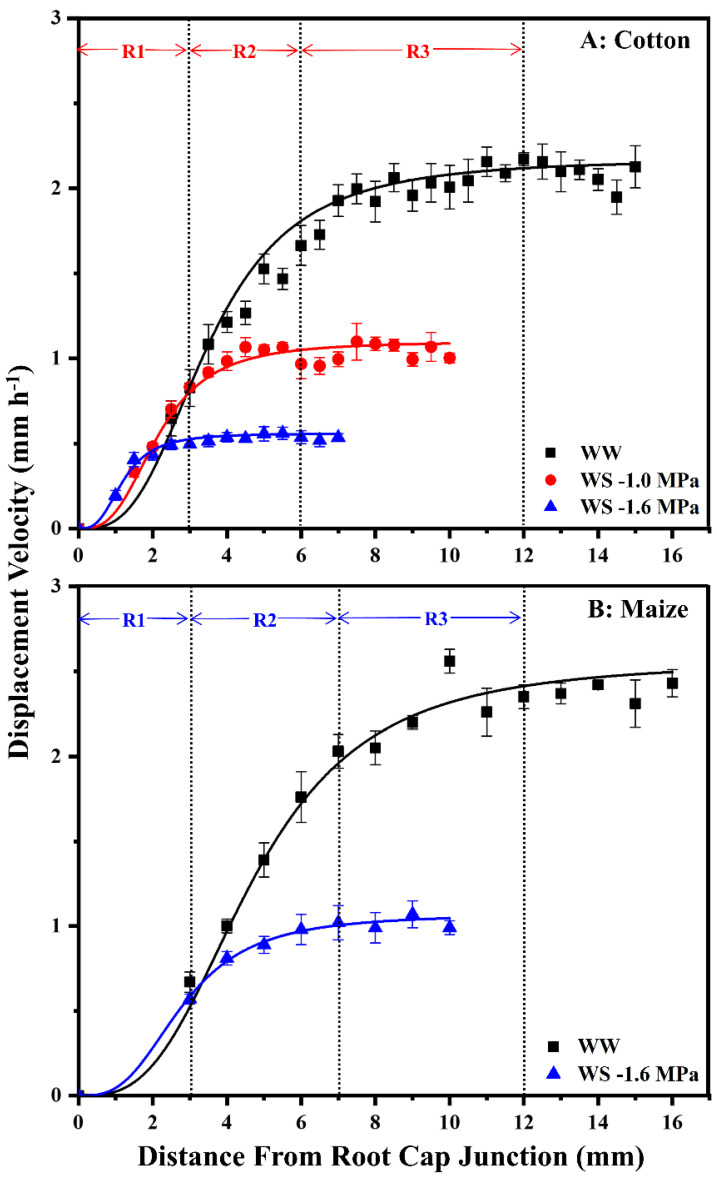
Displacement velocity profiles in the elongation zone of the primary root of cotton (**A**) and maize (**B**) seedlings 48 h after transplanting to well-watered (WW) or water-stressed (WS) conditions. In the water stress treatments, vermiculite water potentials were −1.0 MPa (cotton) and −1.6 MPa (cotton and maize). For each treatment, displacement velocity profiles were calculated for 4–5 individual roots from elongation rates and cell length profiles; values are the means ± SE. R1–3, as described in the text, are indicated for cotton and maize roots in the −1.0 MPa and −1.6 MPa treatments, respectively, which resulted in equivalent root tip water potentials in the two species (Figure 2). The spatial distribution of relative elongation rate is obtained from the derivative of displacement velocity with respect to position. The maize data are reproduced from Sharp et al. [26] with permission from Oxford University Press.

To assess spatial distributions of cell elongation rate and elongation zone lengths, displacement velocity profiles were measured (Figure 3). The displacement velocity describes the velocity of a cell as it passes a certain position during displacement away from the meristem; relative elongation rate profiles are obtained from the derivative of velocity with respect to position. In well-watered roots of both species, elongation initially accelerated, reaching peak rates at 3–4 mm from the root cap junction, followed by progressive deceleration until elongation ceased (i.e., the displacement velocity reached a constant value) at about 12 mm (Figure 3). The effect of water stress on the spatial elongation response in cotton roots was similar to that in maize [3], with maintenance occurring preferentially towards the apex, followed by progressive inhibition and a shortened elongation zone (Figure 3). When cotton and maize roots were grown at equivalent tissue water stress (−1.0 and −1.6 MPa vermiculite water potentials, respectively), displacement velocities increased similarly to those in well-watered roots until nearly 3 mm but were then inhibited and reached constant values at 6–7 mm. When cotton roots were grown under more severe stress at −1.6 MPa, the elongation zone was much further shortened, with the region of maintenance reduced to the apical 1.5 mm (Figure 3A).

Effects on overall cell elongation, as reflected by final cell lengths, and cell flux (the rate at which cells exit the elongation zone) were similar between species (Table S1). Final cell lengths were 85% and 75% of well-watered values for cotton (−1.0 MPa treatment) and maize (−1.6 MPa treatment), respectively, and cell flux was 58% and 52% of well-watered values. Under steady conditions, cell flux approximates the rate of cell production (the rate at which cells leave the meristem). Accordingly, the relative effects of equivalent tissue stress on overall cell elongation versus cell production were similar in the two species, with inhibition of production being more inhibited than elongation. Under more severe tissue stress (−1.6 MPa treatment), cotton roots exhibited further decreases in cell length and cell flux, to 64% and 41% of well-watered values, respectively.

The similar effects of equivalent tissue water stress on cell flux and elongation patterns in cotton and maize primary roots provide the foundation for the comparison of growth regulatory processes. Accordingly, these water deficit treatments, at vermiculite water potentials of −1.0 MPa and −1.6 MPa in cotton and maize, respectively, were used for comparisons of metabolic responses.

### 3.3. Overview of Comparative Metabolomics

Figure 3 illustrates the regions within the cotton and maize primary root elongation zones that were harvested for comparative metabolomics analyses. Region 1 (R1) encompassed the zone of growth maintenance under water stress (0–3 mm, plus the root cap), region 2 (R2) exhibited rapid elongation in well-watered roots but premature deceleration under stress (3–6 mm in cotton, 3–7 mm in maize), and region 3 (R3) comprised the majority of the deceleration zone in well-watered roots. R3 was not analyzed in stressed roots because elongation had ceased.

Water-stressed roots were harvested at 48 h when elongation rates and water potentials were steady (Figure 1 and Figure 2). Comparisons between treatments were filtered to include only changes specifically associated with stress and not with differences in root or cellular development, as follows. Because stressed roots elongated more slowly, two well-watered controls were collected (Figure 1): a developmental control at 24 h and a temporal control at 48 h. Metabolites had to significantly increase or decrease in abundance (and in the same direction) in stressed roots compared with both controls to be regarded as responses to water stress. The kinematic analysis also allowed consideration of effects that could have resulted from differences in cell development profiles between treatments [38]. In R1, elongation rates were the same in stressed and well-watered roots in both species (Figure 3) and, therefore, all significant changes in metabolite abundance were considered responses to stress. To elucidate changes in metabolite abundance associated with growth inhibition in R2, stressed samples were compared with both well-watered R2 and R3. Because R3 of well-watered roots exhibited comparable deceleration to stressed R2 in both species (Figure 3), this allowed the distinction of stress-induced changes from those involved in cell maturation regardless of stress. Accordingly, metabolites that significantly increased or decreased in abundance (and in the same direction) in R2 of stressed roots when compared with both R2 and R3 of well-watered roots were considered potentially associated with stress-induced growth inhibition.

**Figure 4:**
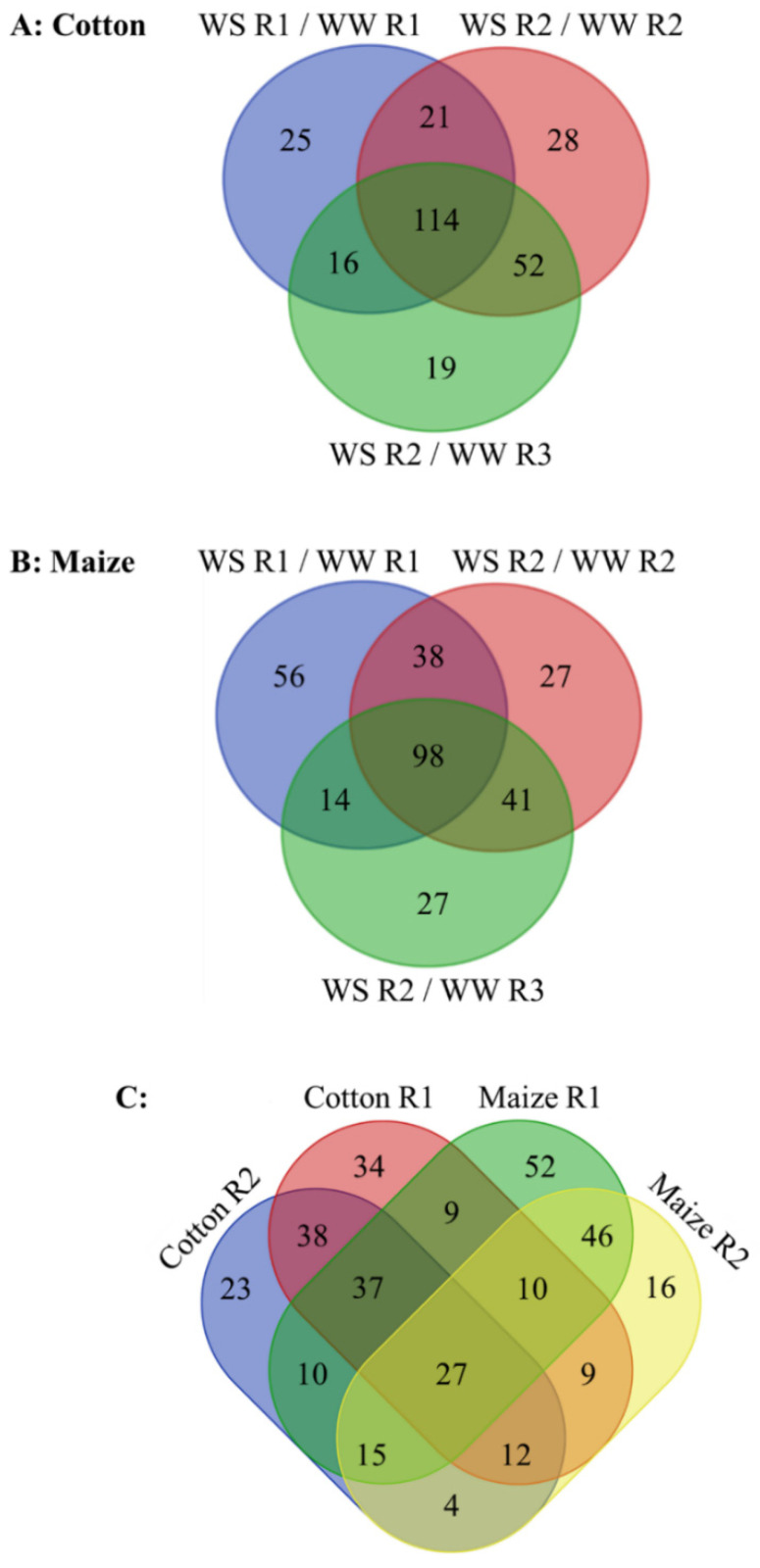
(**A**,**B**) Changes in metabolite abundance in R1 and R2 of the elongation zone of water-stressed (WS) compared with well-watered (WW) cotton and maize primary roots. Changes in water-stressed R2 are also compared with well-watered R3 to help distinguish changes associated with water stress-induced growth inhibition from those associated with cell maturation (see text for details of this analysis). In the water stress treatments, vermiculite water potentials were −1.0 MPa (cotton) and −1.6 MPa (maize), which resulted in equivalent root tip water potentials in the two species (Figure 2). (**C**) Comparison of water stress-induced changes in metabolite abundance in R1 and R2 between cotton and maize primary roots. In R2, only those metabolites that changed in abundance when compared with both R2 and R3 of well-watered roots are included. In all panels, only metabolites that significantly changed in comparison with both the well-watered developmental and temporal controls are included. The metabolites in each category are listed in Supplementary Data S2.

**Figure 5:**
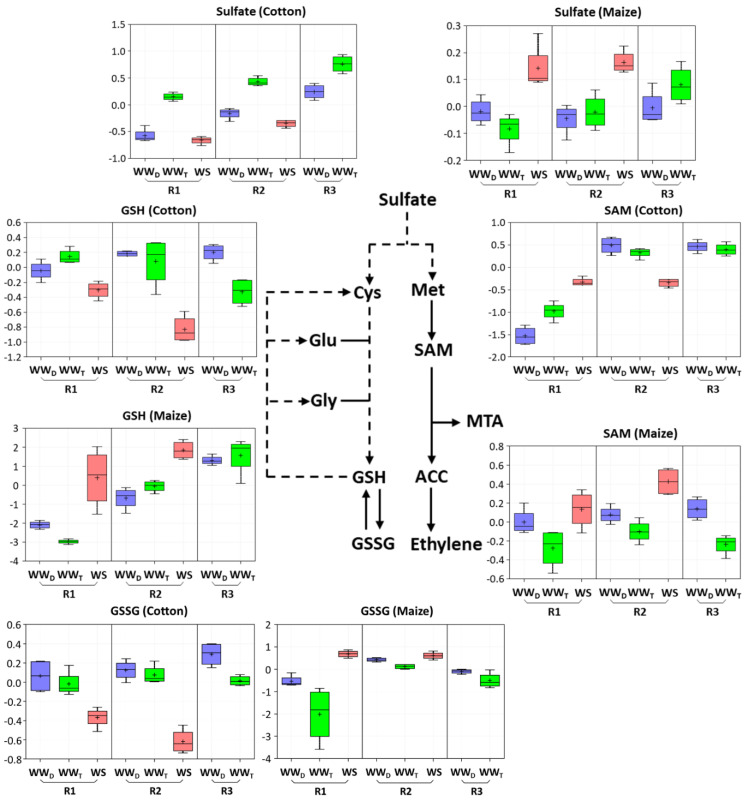
Box plots of the abundance of key metabolites in sulfur and glutathione metabolism in R1 and R2 of the elongation zone of water-stressed (WS) cotton and maize primary roots compared with well-watered developmental (WW_D_) and temporal (WW_T_) controls. R3 of the well-watered controls is also shown to help distinguish changes associated with water stress-induced growth inhibition in R2 from those associated with cell maturation. The box plots represent the natural logarithm (ln) transformation of the scaled data imputed from the raw peak areas of the metabolites. In the water stress treatments, vermiculite water potentials were −1.0 MPa (cotton) and −1.6 MPa (maize), which resulted in equivalent root tip water potentials in the two species (Figure 2). Cys, cysteine; Glu, glutamate; Gly, glycine; Met, methionine.

Metabolomes were assessed using a non-biased, global analysis that identified 342 and 433 metabolites in the cotton and maize root elongation zones, respectively, 92 of which were exclusive to maize while only one, galactinol, was exclusive to cotton (Supplementary Data S1). In total, 275 and 301 metabolites changed in abundance in stressed compared with well-watered cotton and maize roots, respectively (Figure 4; Supplementary Data S2). For cotton, 176 and 166 metabolites changed in abundance in R1 and R2 (when compared with both well-watered R2 and R3), respectively. Of these changes, 41 and 52 were specific to R1 and R2, respectively, and 114 were common to both (Figure 4A). For maize, 206 and 139 metabolites changed in R1 and R2, respectively, with 70 specific to R1, 41 specific to R2, and 98 common to both (Figure 4B). Comparing the species, 34 (R1), 23 (R2), and 38 (both regions) changes were specific to cotton, and 52 (R1), 16 (R2), and 46 (both regions) were specific to maize (Figure 4C). Twenty-one metabolites changed throughout the elongation zone in the same manner in both species, indicating commonalities in the responses, while six metabolites changed in an opposite fashion in R1 compared with R2 in both species (Figure 4C; Table 1). The full range of changes in metabolites for both species is presented in Supplementary Data S1. Major changes that impact the rate of water loss, stress mitigation, or growth for each region are highlighted in Table 1, Table 2 and Table 3 and, in the case of glutathione and sulfur metabolism, Figure 5.

### 3.4. Metabolic Responses to Water Stress in the Whole Elongation Zone

The metabolic changes that occurred in the whole elongation zone (i.e., in both R1 and R2) of water-stressed cotton and maize roots are shown in Table 1. Both species accumulated several sugars in response to stress. Of particular note, cotton exhibited a 27-fold increase in raffinose in R1 and a 72-fold increase in R2, whereas maize exhibited only 10-fold and 7-fold increases, respectively. Maize exhibited a greater increase in sucrose accumulation in R1 compared to R2, whereas cotton exhibited a greater increase in R2 compared to R1.

Both species accumulated proline in R1 and R2 of stressed roots (Table 1). The increase was greater in R1 of maize compared to cotton, but otherwise, the changes were similar. Other notable amino acid-related metabolites that increased in both regions for both species were N-methylproline, pipecolate, saccharopine, and the cysteine derivative taurine. Two important amino acid derivatives, 5-oxoproline and S-carboxymethyl-L-cysteine, significantly decreased in abundance in stressed roots in both species.

The most striking response in the whole elongation zone of maize was the accumulation of the phospholipid glycerophosphorylcholine (GPC), which increased approximately 340-fold in both R1 and R2. In cotton, in contrast, GPC increased by only 2 to 4-fold. Phosphoethanolamine (PE) also increased in abundance in both regions of stressed roots in both species (Table 1).

### 3.5. Metabolic Responses to Water Stress in R1 of the Elongation Zone

The metabolic changes that occurred in R1 of water-stressed cotton and maize roots, where cell elongation was maintained in both species, are shown in Table S2. Amino acid metabolism was substantially altered, with significant differences between cotton and maize as listed in Table 2 and Table 3. In particular, in glutathione and sulfur metabolism, cotton responded with significant decreases (to 0.7-fold) in both reduced (GSH) and oxidized (GSSG) glutathione (Table 3; box plots are shown in Figure 5), as well as similar reductions in 5-oxoproline and the derivatives S-methylglutathione (Table 1) and cysteineglutathione disulfide (Table S2). In striking contrast, R1 of stressed maize roots exhibited 41-fold and 6-fold increases in GSH and GSSG, respectively (Figure 5; Table 3). Maize also showed a significant increase in sulfate that was not evident in cotton (Figure 5; Table 3). Maize exhibited a reduction in 5-oxoproline, but an increase in S-methylglutathione (Table 1).

R1 of water-stressed cotton roots exhibited a significant decline in γ-aminobutyrate (GABA) (Table 1), which derives from α-ketoglutarate (α-KG) via the GABA shunt, along with the accumulation of carboxyethyl-GABA (Table 2). Cotton also accumulated alanine, lysine, methionine, and S-adenosylmethionine (SAM), an important intermediate in methyl group transfer and ethylene biosynthesis, along with the derivative of SAM, 5-methylthioadenosine (MTA) (Figure 5; Table 3 and Table S2). Stressed cotton roots also accumulated serine and betaine (Table 2 and Table S2) to levels 2 to 4-fold higher than in well-watered controls. However, the aromatic amino acid phenylalanine, along with the precursor shikimate, decreased in abundance (Table 1 and Table 2).

Conversely, R1 of maize roots accumulated GABA and exhibited a significant decline in carboxyethyl-GABA in response to water stress (Table 1 and Table 2). R1 also accumulated serine and cysteine (Table 2 and Table 3) along with shikimate, tryptamine, and tyramine (Table S2), and exhibited a 26-fold increase in N-methylphenylalanine and a 0.4-fold decrease in homocysteine that were not detected in cotton (Table S2).

R1 for both species showed a significant decrease in metabolites involved in nucleotide metabolism in response to water stress. The phenylpropanoid pathway was also impacted in both species. In cotton, this was manifested by a significant increase in ferulate (Table 2), whereas in maize, vanillate, 3,4-dimethoxycinnamic acid, 4-hydroxycinnamate and coniferyl aldehyde all increased (Table S2).

### 3.6. Metabolic Responses to Water Stress in R2 of the Elongation Zone

The metabolic changes that occurred in R2 of water-stressed cotton and maize roots, where cell elongation was inhibited in both species, are shown in Table S3. There were several notable differences between cotton and maize. In cotton, as seen for R1, there was a significant decrease in most metabolites associated with glutathione metabolism, including both GSH and GSSG (to 0.45-fold) and sulfate (to 0.5-fold) (Figure 5; Table 3). Glutathione metabolism in R2 of maize, however, was marked only by an increase in S-methylglutathione (Table 1) with no increase in glutathione and a decrease in 5-oxoproline (Table 3). Sulfate levels increased in R2 of maize, as seen for R1, along with an increase in SAM (Figure 5; Table 3).

In the glutamate and aspartate families of amino compounds, R2 of stressed cotton roots exhibited decreased levels of arginine, N-acetylarginine, alanine, SAM, and MTA (Table 2, Table 3 and Table S3), as well as decreases in related compounds including N-acetylasparagine, N-acetylmethionine, and N-formylmethionine (Table S3). Amino acids that increased in R2 of cotton included N-acetylproline, carboxyethyl-GABA, and tryptophan, which did not respond in R1 (Table 2). In maize, apart from amino acids mentioned previously, only serine and quinate increased in abundance in R2 of stressed roots, unlike in R1 (Table 2), whereas tyrosine, phenylalanine, and methionine were decreased (Table 2 and Table 3).

Lipids that accumulated in R2 of stressed roots in both species were similar in level and response to R1 (Table 1, Tables S2 and S3). It is notable that the membrane-bound antioxidant α-tocopherol decreased in region 2 of cotton (Table 2).

### 3.7. Quantitative Assessment of Glutathione Metabolism and Oxidative Stress

**Figure 6:**
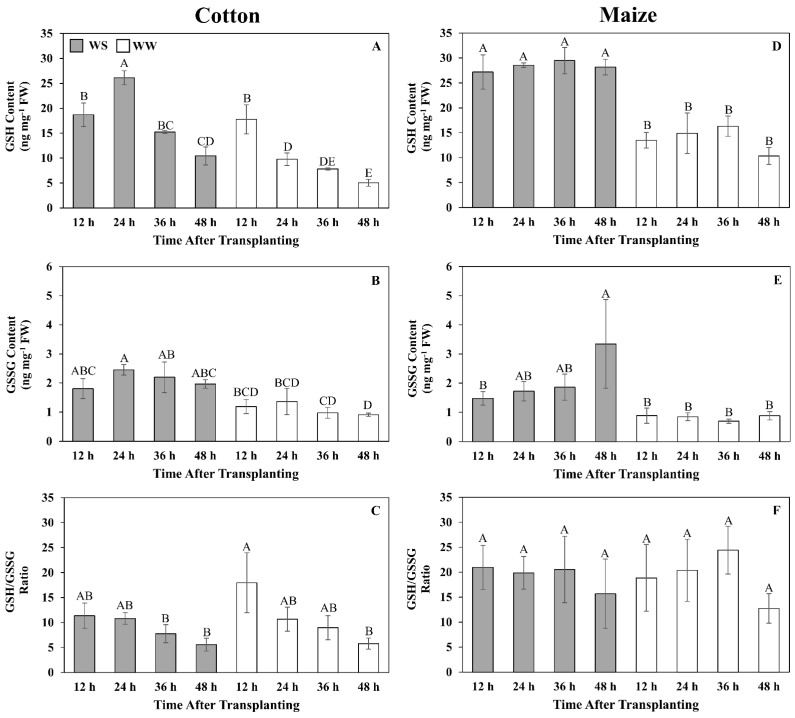
Time courses of reduced (GSH) (**A**,**D**) and oxidized (GSSG) (**B**,**E**) glutathione contents and their ratio (**C**,**F**) in the elongation zone of cotton (**A**–**C**) and maize (**D**–**F**) primary roots during 48 h after transplanting to water-stressed (WS, shaded bars) or well-watered (WW, open bars) conditions. In the water stress treatments, vermiculite water potentials were −1.0 MPa (cotton) and −1.6 MPa (maize), which resulted in equivalent root tip water potentials in the two species (Figure 2). The whole elongation zone (water-stressed cotton, 0–6 mm; water-stressed maize, 0–7 mm; well-watered cotton and maize, 0–12 mm) was collected. Data are the means ± SE (*n* = 3–6). ANOVA analyses compare the data at different time points across treatments within each species; different letters indicate significant differences (*p* < 0.05).

The unbiased global metabolomics indicated that cotton and maize primary root elongation zones significantly differed in their control and mitigation strategies for water stress-induced oxidative stress, especially with regard to glutathione metabolism. As detailed above, glutathione and associated metabolites declined throughout the elongation zone in stressed cotton roots, whereas in R1 of maize roots, both glutathione and associated metabolites increased significantly (Figure 5; Table 3). To quantify glutathione levels and examine the time course of the differential responses between the species, GSH and GSSG were measured using an enzyme-based assay [30]. Given tissue requirements and the decrease in glutathione levels throughout the elongation zone in cotton, whole elongation zone samples were assayed (Figure 6).

In well-watered cotton, GSH was relatively high, at 18 ng mg^−1^ FW, at 12 h but declined steadily to reach 5 ng mg^−1^ FW after 48 h (Figure 6A). GSSG was relatively stable at approximately 1 ng mg^−1^ FW throughout the growth period (Figure 6B). In water-stressed cotton, GSH levels were similar to those of well-watered roots at 12 h but were elevated to 27 ng mg^−1^ FW at 24 h, after which levels markedly declined but remained above well-watered values (Figure 6A). GSSG levels were slightly higher than in well-watered roots, at approximately 2 ng mg^−1^ FW, and relatively stable throughout the 48 h period (Figure 6B). The GSH/GSSG ratio in both well-watered and stressed cotton roots exhibited similar kinetics in that the ratio was highest at 12 h and then steadily declined to approximately 5:1 (Figure 6C).

In well-watered maize, both GSH and GSSG levels were relatively stable at approximately 15 and 1 ng mg^−1^ FW, respectively. GSH levels were markedly elevated in response to water stress, reaching almost 30 ng mg^−1^ FW throughout the 48 h period (Figure 6D). GSSG levels also steadily increased after transplanting to water stress, reaching 3.2 ng mg^−1^ FW at 48 h (Figure 6E). The ratio of GSH/GSSG did not change significantly throughout the 48 h period for both well-watered and stressed roots, averaging approximately 20:1 in both treatments (Figure 6F).

**Figure 7:**
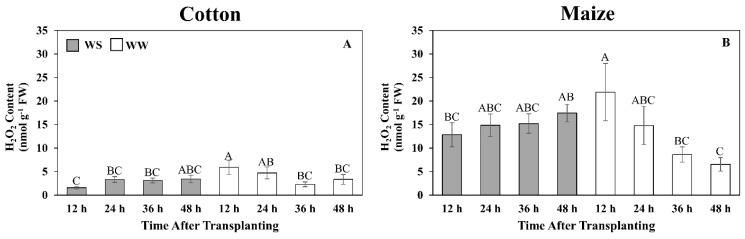
Time courses of hydrogen peroxide (H_2_O_2_) content in the elongation zone of cotton (**A**) and maize (**B**) primary roots during 48 h after transplanting to water-stressed (WS, shaded bars) or well-watered (WW, open bars) conditions. In the water stress treatments, vermiculite water potentials were −1.0 MPa (cotton) and −1.6 MPa (maize), which resulted in equivalent root tip water potentials in the two species (Figure 2). The whole elongation zone was collected (see Figure 6 for dimensions). Data are the means ± SE (*n* = 6). ANOVA analyses compare the data at different time points across treatments within each species; different letters indicate significant differences (*p* < 0.05).

Although the quantitative glutathione measurements indicate that the cotton root elongation zone did exhibit a glutathione response following exposure to water stress, which was not revealed in the 48 h metabolomics single time-point assessment, it was clearly less in magnitude and duration than in maize. To investigate the impact of this differential response on the level of oxidative stress, the time course of H_2_O_2_ content was assayed under well-watered and water-stressed conditions (Figure 7). In cotton, H_2_O_2_ levels remained relatively low (6 nmol g^−1^ FW or less) in both treatments, with a significant difference apparent only at 12 h, when levels were lower in the stressed roots (Figure 7A). In well-watered maize roots, H_2_O_2_ levels were highest at 12 h (approximately 23 nmol g^−1^ FW) and then declined to 6 nmol g^−1^ FW after 48 h. In contrast, in response to water stress, H_2_O_2_ levels gradually increased to 17 nmol g^−1^ FW after 48 h (Figure 7B).

**Figure 8:**
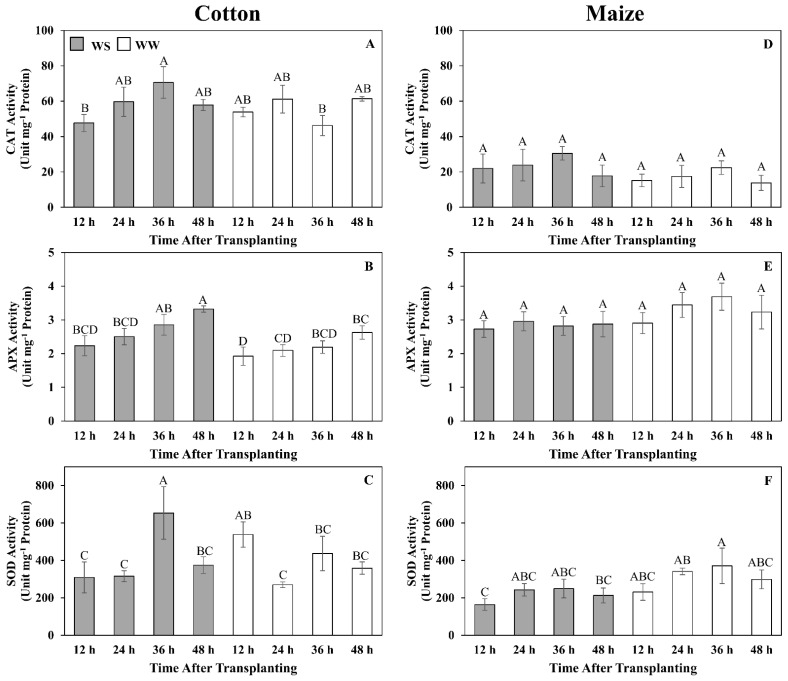
Time courses of catalase (CAT) (**A**,**D**), ascorbate peroxidase (APX) (**B**,**E**) and superoxide dismutase (SOD) (**C**,**F**) activities in the elongation zone of cotton (**A**–**C**) and maize (**D**–**F**) primary roots during 48 h after transplanting to water-stressed (WS, shaded bars) or well-watered (WW, open bars) conditions. In the water stress treatments, vermiculite water potentials were −1.0 MPa (cotton) and −1.6 MPa (maize), which resulted in equivalent root tip water potentials in the two species (Figure 2). The whole elongation zone was collected (see Figure 6 for dimensions). Data are the means ± SE (*n* = 4). ANOVA analyses compare the data at different time points across treatments within each species; different letters indicate significant differences (*p* < 0.05).

With the much lower H_2_O_2_ contents in cotton under both well-watered and water-stressed conditions, we explored the possibility that cotton had a more efficient enzymatic defense against H_2_O_2_ accumulation relative to maize. The time course of activities for three primary oxidative enzymes, CAT, APX, and SOD were determined (Figure 8). In cotton and maize roots, CAT activities were relatively stable and not significantly different between treatments (except at 36 h in cotton when activity was higher in stressed roots) (Figure 8A,D). However, CAT activities were approximately three-fold higher in cotton than in maize. In cotton, APX activity increased gradually in both treatments and was significantly higher in stressed roots by 48 h (Figure 8B), whereas in maize, APX activity was stable and not significantly different between treatments (Figure 8E). SOD activities were the most variable of the three enzymes in both treatments of both species but were generally higher in cotton than in maize (Figure 8C,F).

## 4. Discussion

**Figure 9:**
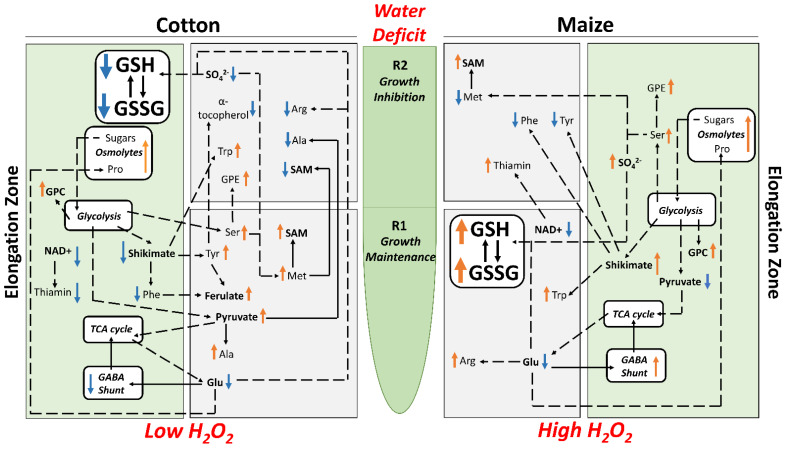
Summary of key changes in metabolite abundance in R1 and R2 and the whole elongation zone of cotton (**left** panels) and maize (**right** panels) primary roots growing under water deficit conditions with a focus on metabolites related to osmotic adjustment, antioxidative metabolism, sulfur metabolism and ultimately growth. Arrows adjacent to the metabolites indicate increases (orange) or decreases (blue) in abundance in water-stressed compared with well-watered roots.

By imposing external water-deficit conditions that generated equivalent tissue water potentials (approximately −1.6 MPa) in the elongation zones of water-stressed cotton and maize primary roots, we demonstrated that cotton exhibits growth characteristics that closely match those of maize for both cell elongation and proliferation (Figure 3; Table S1). It is precisely because this approach delivered equivalent tissue water stress levels and root growth characteristics in both species that it allowed for direct metabolic comparisons of how strategies of stress mitigation and growth regulation interact in each species to generate regions of growth maintenance (R1) and inhibition (R2). Metabolomics and quantitative analyses demonstrated that these responses were achieved by different metabolic strategies in the two species, which are summarized in Figure 9.

### 4.1. Reducing Water Loss during Exposure to Low Water Potentials

Osmotic adjustment (osmolyte accumulation) is a critical response to low water potentials that reduces water loss by lowering cellular osmotic potential [42] and is important for maintaining root growth under water stress [43,44,45]. Osmolyte accumulation occurred in both the regions of growth maintenance and inhibition in water-stressed cotton and maize roots, consistent with roles in maintaining hydration and viability as well as cell expansion (Figure 9). In both species, sucrose and proline as well as several other carbohydrates and amino acids increased in abundance. Proline accumulation in response to water stress is common [46,47,48], and in addition to serving as an osmolyte, can function as an oxidative protectant to maintain membrane integrity and stabilize proteins, and can interact with hormones including abscisic acid to enhance stress tolerance [49,50,51]. Levels of pipecolate, which shares a common biosynthetic pathway with proline, increased within the whole elongation zone of both species and may play similar roles to proline [52]. Raffinose, which accumulated substantially in both species but particularly in cotton, can also serve as an antioxidant [53,54].

Less clear is the role that increased levels of two phospholipids, PE and GPC, play in the response to water stress, in particular the much greater accumulation of GPC throughout the elongation zone of maize than cotton. Accumulation of glycerophospholipids could be indicative of stress-induced damage, signaling plasma membrane degradation and remodeling [55]. It is also possible that PE and GPC (a precursor to phosphatidylcholine) increase membrane flexibility [56]. Glycerophospholipids, especially GPC, could also participate in osmotic adjustment and anti-oxidative metabolism [57].

### 4.2. Metabolic Responses to Water Stress Associated with Growth

The flow of nitrogen and carbon into the deposition of osmolytes in the elongation zone likely contributes to the overall suppression of growth, although in R1 where elongation is maintained in both species this must be compensated for in some way. It is possible that for R1 these compounds derive from elsewhere in the plant and water stress may enhance phloem transport of sugars [58] and proline [59] to the root tip.

Shikimate, the precursor of aromatic amino acids, decreased in abundance in the cotton elongation zone but increased in maize, and these responses were associated with diverse responses of aromatic amino acids and their derivatives (Figure 9). Tyrosine accumulated in R1 of cotton but decreased in R2 of maize, while phenylalanine decreased in abundance in the whole growth zone of cotton but only in R2 of maize. These changes were also reflected in the accumulation of ferulate and coniferyl aldehyde, metabolites that are downstream of phenylalanine, in R1 of cotton and maize, respectively. These metabolites are derived from 4-hydroxycinnamate, which showed opposite changes in R1 of cotton and maize. Although maize did not exhibit a significant change in ferulate abundance, it did accumulate the derivative vanillate in R1, which may indicate robust biosynthesis and utilization of ferulate in maize. These phenylpropanoids are important secondary metabolites that may function in the regulation of cell wall expansion under water-stressed conditions [7].

Cotton and maize roots also differed in metabolite pools associated with sulfur metabolism, which can play a role in both growth and water stress responses (Figure 9). Declines in both sulfate and glutathione throughout the elongation zone in water-stressed cotton appear to drive an increase in SAM and MTA. In maize, in contrast, sulfur metabolism appeared to focus on maintaining elevated levels of glutathione. A major function of sulfur metabolism under water stress appears related to the production of the sulfur-containing amino acids cysteine and methionine and their derivatives, including glutathione and SAM, respectively [60]. The differential accumulations of glutathione and SAM in cotton and maize roots may reflect competing interests of the sulfur resource when exposed to water stress. A byproduct of MTA biosynthesis via SAM is 1-aminocyclopropane-1-carboxylic acid (ACC), the substrate for ethylene production (Figure 5), which is involved in both water stress resistance and growth regulation [61,62]. SAM is also the precursor of some polyamines that act as osmoprotectants [63,64]. Unfortunately, the metabolomics study did not uncover ACC or provide a comprehensive assessment of polyamine changes for either species.

In general, metabolites associated with the TCA cycle decreased in both regions of water-stressed roots in both species, indicating that this central pathway may have been taxed (Figure 9). However, there were dissimilarities between maize and cotton, e.g., decreased fumarate in cotton and increased α-KG in maize, which point to differences in the flux of compounds through the pathway [65] and perhaps different metabolic priorities for the TCA cycle. This is also suggested by the response of the GABA shunt, a closed branch pathway of the TCA cycle. GABA accumulates to high levels in plants exposed to abiotic stresses including drought [66] and was elevated in the maize elongation zone in response to water stress. However, GABA declined in both regions in cotton. The GABA shunt and GABA function in pH homeostasis [66] and antioxidant metabolism [67]. It is possible that cotton and maize roots have different pH requirements to establish the responses of the elongation zone to water-stressed conditions.

The cofactor NAD+, a central metabolite of redox metabolism [68], was reduced in the whole elongation zone of cotton and R1 of maize, which is perhaps antithetical to its suggested role in stress-induced proline and ABA biosynthesis [69]. The GABA shunt converts NAD+ to NADH required for the glutathione cycle [66,70]. In water-stressed cotton roots, the demand for NADH may not be high as the glutathione cycle was repressed. Such was not the case for maize, where GABA accumulated and NAD+ levels were only reduced in R1.

### 4.3. Antioxidative Metabolism

Glutathione metabolism is also central to redox metabolism and homeostasis [71], and glutathione has generally been observed to accumulate in response to water stress [72], including in maize roots [48]. However, to the best of our knowledge, glutathione levels have not been quantitatively assessed in the cotton primary root elongation zone. At the single time point (48 h) used for the metabolomic analysis, GSH and GSSG declined in response to water stress in both regions of the cotton elongation zone but increased significantly in R1 of maize. The quantitative time-course analysis of glutathione levels in large part corroborated the differential response of glutathione metabolism between the species. Although cotton roots did exhibit an elevated glutathione response to water stress, it occurred primarily in the first 24 h and then GSH levels dramatically declined, whereas in maize, glutathione levels were markedly increased throughout the 48 h stress exposure (GSH) or increased with time (GSSG) compared with well-watered controls. Notably, the glutathione precursor 5-oxoproline was significantly reduced in both cotton and maize. In cotton, this may indicate inhibition of the glutathione cycle per se, whereas in maize, it may reflect increased synthesis of glutathione (Figure 9).

The increased levels of GSH and GSSG in R1 of the elongation zone of maize roots demonstrate that maize utilizes the glutathione cycle to regulate ROS levels. In addition to countering the negative impacts of ROS [73], glutathione can act as a signaling molecule [74]. Several studies have shown that glutathione levels and the sub-cellular GSH/GSSG ratio can regulate metabolic processes, including cell cycle progression [74,75,76]. In this context, it is interesting to note that an R1-specific increase in apoplastic ROS was observed in water-stressed maize primary roots [38,40], and this increase was shown to be involved in the regulation of cell production and root elongation [77]. It is tempting to speculate that the increased levels of both GSH and GSSG in R1 of water-stressed maize primary roots are involved in transmitting the ROS signal and in the regulation of cell production.

In contrast to the responses in maize, the decrease in GSH and associated decline in the GSH:GSSG ratio in the cotton root elongation zone as exposure to water stress progressed could suggest a deficiency in the ability to counter stress-induced elevations in ROS, or that high levels of ROS reduced glutathione levels as seen in canola [78]. Lower ratios of GSH:GSSG have been considered indicative of exposure to high levels of oxidative stress [73]. However, H_2_O_2_ levels in water-stressed cotton roots remained low, whereas in maize, which maintained a relatively high GSH:GSSG ratio in both treatments, H_2_O_2_ levels were significantly higher in water-stressed compared to well-watered roots by the end of the experiments. Consistent with the cotton root experiencing only low ROS levels during water stress, other antioxidants including GABA and alpha-tocopherol, which inhibit lipid peroxidation [79], also decreased in abundance. The glutathione, ROS, and GABA/NAD+ data, taken together, indicate that the similar growth responses of water-stressed cotton and maize roots were derived in the context of different redox states.

It seems unlikely that ROS were not generated in the cotton root elongation zone under water-stressed conditions, suggesting that cotton may employ a more effective enzymatic anti-oxidation defense than maize. Activities of three major anti-oxidative enzymes, CAT, APX, and SOD, have been reported to be water stress-induced in both cotton and maize [38,47,80], although in this study, only APX exhibited stress induction and only in cotton. However, the results show that the cotton root elongation zone had inherently higher CAT and SOD activities that, along with the increase in APX activity, likely contributed to a greater ability to prevent a toxic buildup of ROS during water stress.

## 5. Conclusions

Under severe water stress, the cotton and maize primary root elongation zones exhibited similar spatial patterns of cell elongation and cell flux at equivalent tissue water potentials. Metabolomics and quantitative analyses showed region-specific and species-specific changes in metabolite abundance in response to water stress, revealing both conserved responses including osmolyte accumulation, and key differences including variations in metabolite pools associated with sulfur metabolism, which can play important roles in both growth and water stress responses. Redox metabolism, in particular, involving both antioxidants and antioxidant enzymatic activities, characterized differing metabolic responses underlying the establishment of zones of growth maintenance and inhibition in each species.

## Figures and Tables

**Table 1:** Ratios of metabolite abundance between water-stressed and well-watered primary roots of cotton and maize for metabolites showing consistent responses in R1 and R2 of the elongation zone.

Super Pathway	Metabolite	R1		R2	
		Cotton	Maize	Cotton	Maize
**Carbohydrate**	Raffinose	**26.76**	**10.42**	**71.6**	**6.97**
	Sucrose	**4.62**	**11.95**	**17.33**	**4.88**
	Fructose	**3.26**	**2.61**	**1.81**	**2.3**
	Glucose	**2.9**	**1.77**	**1.71**	**1.35**
	Myo-inositol	**2.59**	**1.45**	**2.11**	**1.75**
**Lipids**	GPC	**2.27**	**338.42**	**3.74**	**342.28**
	Phosphoethanolamine	**3.66**	**6.49**	**1.74**	**2.67**
**Amino acid**	Saccharopine	**8.9**	**19.51**	**4.13**	**16.39**
	Pipecolate	**8.76**	**3.63**	**5.05**	**4.02**
	Proline	**5.62**	**16.34**	**5.04**	**4.63**
	N-methylproline	**2.4**	**2.99**	**2.37**	**3.6**
	Taurine	**3.67**	**3**	**3.97**	**1.95**
	β-hydroxyisovalerate	**5.05**	**0.49**	**3.43**	**0.46**
	N6-acetyllysine	**3.75**	**0.34**	**2.28**	**0.52**
	2-aminoadipate	**2.49**	**0.11**	**1.9**	**0.28**
	S-carboxymethyl-L-cysteine	**0.64**	**0.26**	**0.62**	**0.4**
	5-oxoproline	**0.64**	**0.53**	**0.39**	**0.37**
	Glutamine	**0.54**	**0.52**	**0.2**	**0.36**
	Histidine	**0.53**	**0.61**	**0.48**	**0.65**
	N-formylphenylalanine	**0.39**	**0.09**	**0.4**	**0.11**
	GABA	**0.69**	**2.79**	**0.54**	**3.97**
	Shikimate	**0.51**	**1.95**	**0.33**	**4.89**
	S-methylglutathione	**0.49**	**2.45**	**0.63**	**1.99**
**Others**	Threonate	**0.77**	**0.57**	**0.73**	**0.69**
	Riboflavin	**0.69**	**0.5**	**0.45**	**0.67**
	Adenosine	**0.55**	**0.61**	**0.45**	**0.68**
	Xanthosine	**0.27**	**0.35**	**0.39**	**0.28**

In the water stress treatments, vermiculite water potentials were −1.0 MPa (cotton) and −1.6 MPa (maize), which resulted in equivalent root tip water potentials in the two species (Figure 2). In R1, the fold-changes are the averages of comparisons of the water-stressed treatment to well-watered developmental and temporal controls. In R2, the fold-changes are the averages of comparisons of the water-stressed treatment to the well-watered controls in both R2 and R3 (see text for details of this analysis). Yellow and blue cells indicate significant increases or decreases in abundance, respectively (darker shades, *p* < 0.05 in all comparisons; lighter shades, 0.05 < *p* < 0.10 in at least one comparison). No minimum cutoff value was applied to the fold changes.

**Table 2:** Ratios of abundance of representative metabolites between water-stressed and well-watered primary roots of cotton and maize for metabolites showing specific responses in R1 or R2 of the elongation zone in at least one species.

Super Pathway	Metabolite	R1		R2	
		Cotton	Maize	Cotton	Maize
**Carbohydrate**	Pyruvate	**1.94**	**0.53**	**NS**	**0.6**
	Malate	**NS**	**0.67**	**0.5**	**NS**
**Amino acid**	N-acetylproline	**4.65**	**1.75**	**5.04**	**NS**
	Serine	**1.76**	**2.58**	**NS**	**1.82**
	Carboxyethyl-GABA	**1.92**	**0.41**	**1.64**	**NS**
	Tyrosine	**1.35**	**NS**	**NS**	**0.5**
	Arginine	**NS**	**1.47**	**0.47**	**NS**
	Tryptophan	**NS**	**1.55**	**1.58**	**NS**
	Glutamate	**0.72**	**0.71**	**NS**	**NS**
	Quinate	**0.5**	**NS**	**0.51**	**2.16**
	Phenylalanine	**0.7**	**NS**	**0.42**	**0.65**
**Lipids**	GPE	**NS**	**31.92**	**1.87**	**12.27**
	1-palmitoyl-GPG (16:0)	**0.62**	**NS**	**0.38**	**2.92**
**Others**	2’-AMP	**1.6**	**NS**	**NS**	**0.53**
	IMP	**2.32**	**0.6**	**0.36**	**NS**
	Ferulate	**1.76**	**NS**	**NS**	**NS**
	Allantoin	**NS**	**3.04**	**0.59**	**1.52**
	FAD	**0.51**	**0.35**	**0.49**	**NS**
	Thiamin	**0.51**	**NS**	**0.56**	**2.29**
	α-tocopherol	**NS**	**NS**	**0.51**	**NS**

In the water stress treatments, vermiculite water potentials were −1.0 MPa (cotton) and −1.6 MPa (maize), which resulted in equivalent root tip water potentials in the two species (Figure 2). Yellow and blue cells indicate significant increases or decreases in abundance, respectively (darker shades, *p* < 0.05 in all comparisons; lighter shades, 0.05 < *p* < 0.10 in at least one comparison). White cells indicate non-significant (NS) changes in at least one comparison. See Table 1 for other details.

**Table 3:** Ratios of abundance of metabolites involved in sulfur and glutathione metabolism in R1 and R2 of the elongation zone of water-stressed compared with well-watered primary roots of cotton and maize.

Metabolite	Cotton		Maize	
	R1	R2	R1	R2
5-oxoproline	**0.64**	**0.39**	**0.53**	**0.37**
Glutathione, oxidized (GSSG)	**0.68**	**0.48**	**6.4**	**NS**
Glutathione, reduced (GSH)	**0.71**	**0.43**	**41.38**	**NS**
γ-glutamylleucine	**2.5**	**1.43**	**NS**	**NS**
γ-glutamylthreonine	**NS**	**NS**	**1.87**	**2.75**
Ophthalmate	**4.42**	**NS**	**NS**	**NS**
Norophthalmate			**1.64**	**NS**
Cys-gly, oxidized	**2.54**	**NS**	**4.85**	**7.07**
Sulfate	**NS**	**0.54**	**1.22**	**1.18**
Cysteine	**NS**	**NS**	**1.7**	**NS**
Methionine	**3.08**	**NS**	**NS**	**0.31**
S-adenosylmethionine (SAM)	**2.59**	**0.47**	**NS**	**1.61**
5-methylthioadenosine (MTA)	**1.56**	**0.6**	**NS**	**NS**

In the water stress treatments, vermiculite water potentials were −1.0 MPa (cotton) and −1.6 MPa (maize), which resulted in equivalent root tip water potentials in the two species (Figure 2). Yellow and blue cells indicate significant increases or decreases in abundance, respectively (darker shades, *p* < 0.05 in all comparisons; lighter shades, 0.05 < *p* < 0.10 in at least one comparison). White cells indicate non-significant (NS) changes in at least one comparison; gray cells indicate that the metabolite was not detected. See Table 1 for other details.

## Data Availability

Data are contained within the article and Supplementary Materials.

## Supplementary Materials

The following supporting information can be downloaded at: https://www.mdpi.com/article/10.3390/antiox11050820/s1. Figure S1: Cotton primary root elongation in non-sieved and sieved vermiculite at a water potential of −1.6 MPa. Table S1: Root growth characteristics of cotton and maize primary roots under well-watered and water-stressed conditions. Table S2: Ratios of metabolite abundance in R1 of the elongation zone between water-stressed and well-watered primary roots of cotton and maize. Table S3: Ratios of metabolite abundance in R2 of the elongation zone between water-stressed and well-watered primary roots of cotton and maize. Supplementary Data S1: Metabolite profiles of different regions within the elongation zone of cotton and maize primary roots in well-watered developmental control, well-watered temporal control, and water-stressed treatments. Supplementary Data S2: Identities of metabolites in the different categories shown in Figure 4.

## References

[1] Zhang H., Zhao Y., Zhu J.-K. (2020). Thriving under stress: How plants balance growth and the stress response. Dev. Cell.

[2] Ober E.S., Sharp R.E., Eshel A., Beekman T. (2013). Maintaining root growth in drying soil: A review of progress and gaps in understanding. Plant Roots: The Hidden Half.

[3] Sharp R.E., Silk W.K., Hsiao T.C. (1988). Growth of the maize primary root at low water potentials. I. Spatial distribution of expansive growth. Plant Physiol..

[4] Spollen W.G., Sharp R.E., Saab I.N., Wu Y., Smith J.A.C., Griffiths H. (1993). Regulation of cell expansion in roots and shoots at low water potentials. Water Deficits: Plant Responses from Cell to Community.

[5] van der Weele C.M., Spollen W.G., Sharp R.E., Baskin T.I. (2000). Growth of *Arabidopsis thaliana* seedlings under water deficit by control of water potential in nutrient-agar media. J. Exp. Bot..

[6] Yamaguchi M., Valliyodan B., Zhang J., LeNoble M.E., Yu O., Rogers E.E., Nguyen H.T., Sharp R.E. (2010). Regulation of growth response to water stress in the soybean primary root. I. Proteomic analysis reveals region-specific regulation of phenylpropanoid metabolism and control of free iron in the elongation zone. Plant Cell Environ..

[7] Yamaguchi M., Sharp R.E. (2010). Complexity and coordination of root growth at low water potentials: Recent advances from transcriptomic and proteomic analyses. Plant Cell Environ..

[8] Rosenow D.T., Quisenberry J.E., Wendt C.W., Clark L.E. (1983). Drought tolerant sorghum and cotton germplasm. Agric. Water Manag..

[9] Chapagain A.K., Hoekstra A.Y., Savenije H.H.G., Gautam R. (2006). The water footprint of cotton consumption: An assessment of the impact of worldwide consumption of cotton products on the water resources in the cotton producing countries. Ecol. Econ..

[10] Esmaeili N., Cai Y., Tang F., Zhu X., Smith J., Mishra N., Hequet E., Ritchie G., Jones D., Shen G. (2021). Towards doubling fibre yield for cotton in the semiarid agricultural area by increasing tolerance to drought, heat and salinity simultaneously. Plant Biotechnol. J..

[11] Liu S., Remley M., Bourland F.M., Nichols R.L., Stevens W.E., Phillips Jones A., Fritschi F.B. (2015). Early vigor of advanced breeding lines and modern cotton cultivars. Crop Sci..

[12] Virk G., Snider J.L., Pilon C. (2019). Physiological contributors to early season whole-crop vigor in cotton. Crop Sci..

[13] Klepper B., Taylor H.M., Huck M.G., Fiscus E.L. (1973). Water relations and growth of cotton in drying soil. Agron. J..

[14] McMichael B.L., Quisenberry J.E. (1991). Genetic variation for root-shoot relationships among cotton germplasm. Environ. Exp. Bot..

[15] Cook C.G., El-Zik K.M. (1992). Cotton seedling and first-bloom plant characteristics: Relationships with drought-influenced boll abscission and lint yield. Crop Sci..

[16] Basal H., Smith C.W., Thaxton P.S., Hemphill J.K. (2005). Seedling drought tolerance in upland cotton. Crop Sci..

[17] Taylor H.M., Ratliff L.F. (1969). Root elongation rates of cotton and peanuts as a function of soil strength and soil water content. Soil Sci..

[18] Pace P.F., Cralle H.T., El-Halawany S.H.M., Cothren J.T., Senseman S.A. (1999). Drought-induced changes in shoot and root growth of young cotton plants. J. Cotton Sci..

[19] Bowne J.B., Erwin T.A., Juttner J., Schnurbusch T., Langridge P., Bacic A., Roessner U. (2012). Drought responses of leaf tissues from wheat cultivars of differing drought tolerance at the metabolite level. Mol. Plant.

[20] Hochberg U., Degu A., Toubiana D., Gendler T., Nikoloski Z., Rachmilevitch S., Fait A. (2013). Metabolite profiling and network analysis reveal coordinated changes in grapevine water stress response. BMC Plant Biol..

[21] Boyer J.S., Knipling E.B. (1965). Isopiestic technique for measuring leaf water potentials with a thermocouple psychrometer. Proc. Natl. Acad. Sci. USA.

[22] Saab I.N., Sharp R.E., Pritchard J., Voetberg G.S. (1990). Increased endogenous abscisic acid maintains primary root growth and inhibits shoot growth of maize seedlings at low water potentials. Plant Physiol..

[23] Verslues P.E., Ober E.S., Sharp R.E. (1998). Root growth and oxygen relations at low water potentials: Impact of oxygen availability in polyethylene glycol solutions. Plant Physiol..

[24] Nonami H., Boyer J.S. (1989). Turgor and growth at low water potentials. Plant Physiol..

[25] Silk W.K., Lord E.M., Eckard K.J. (1989). Growth patterns inferred from anatomical records: Empirical tests using longisections of roots of *Zea mays* L.. Plant Physiol..

[26] Sharp R.E., Poroyko V., Hejlek L.G., Spollen W.G., Springer G.K., Bohnert H.J., Nguyen H.T. (2004). Root growth maintenance during water deficits: Physiology to functional genomics. J. Exp. Bot..

[27] Erickson R.O. (1961). Probability of division of cells in the epidermis of the *Phleum* root. Am. J. Bot..

[28] Evans A.M., DeHaven C.D., Barrett T., Mitchell M., Milgram E. (2009). Integrated, nontargeted ultrahigh performance liquid chromatography/electrospray ionization tandem mass spectrometry platform for the identification and relative quantification of the small-molecule complement of biological systems. Anal. Chem..

[29] Yobi A., Wone B.W.M., Xu W., Alexander D.C., Guo L., Ryals J.A., Oliver M.J., Cushman J.C. (2013). Metabolomic profiling in *Selaginella lepidophylla* at various hydration states provides new insights into the mechanistic basis of desiccation tolerance. Mol. Plant.

[30] Noctor G., Mhamdi A., Foyer C.H. (2016). Oxidative stress and antioxidative systems: Recipes for successful data collection and interpretation. Plant Cell Environ..

[31] Le C.T.T., Brumbarova T., Ivanov R., Stoof C., Weber E., Mohrbacher J., Fink-Straube C., Bauer P. (2016). Zinc finger of arabidopsis thaliana12 (ZAT12) interacts with fer-like iron deficiency-induced transcription factor (FIT) linking iron deficiency and oxidative stress responses. Plant Physiol..

[32] Bradford M.M. (1976). A rapid and sensitive method for the quantitation of microgram quantities of protein utilizing the principle of protein-dye binding. Anal. Biochem..

[33] Aebi H. (1984). Catalase in vitro. Meth. Enzymol..

[34] Amako K., Chen G.-X., Asada K. (1994). Separate assays specific for ascorbate peroxidase and guaiacol peroxidase and for chloroplastic and cytosolic isozymes of ascorbate peroxidase in plants. Plant Cell Physiol..

[35] Giannopolitis C.N., Ries S.K. (1977). Superoxide dismutases: I. Occurrence in higher plants. Plant Physiol..

[36] Leach K.A., Hejlek L.G., Hearne L.B., Nguyen H.T., Sharp R.E., Davis G.L. (2011). Primary root elongation rate and abscisic acid levels of maize in response to water stress. Crop Sci..

[37] Spollen W.G., Tao W., Valliyodan B., Chen K., Hejlek L.G., Kim J.J., LeNoble M.E., Zhu J., Bohnert H.J., Henderson D. (2008). Spatial distribution of transcript changes in the maize primary root elongation zone at low water potential. BMC Plant Biol..

[38] Zhu J., Alvarez S., Marsh E.L., LeNoble M.E., Cho I.J., Sivaguru M., Chen S., Nguyen H.T., Wu Y., Schachtman D.P. (2007). Cell wall proteome in the maize primary root elongation zone. II. Region-specific changes in water soluble and lightly ionically bound proteins under water deficit. Plant Physiol..

[39] Voothuluru P., Anderson J.C., Sharp R.E., Peck S.C. (2016). Plasma membrane proteomics in the maize primary root growth zone: Novel insights into root growth adaptation to water stress. Plant Cell Environ..

[40] Voothuluru P., Sharp R.E. (2013). Apoplastic hydrogen peroxide in the growth zone of the maize primary root under water stress. I. Increased levels are specific to the apical region of growth maintenance. J. Exp. Bot..

[41] Seeve C.M., Cho I.-J., Hearne L.B., Srivastava G.P., Joshi T., Smith D.O., Sharp R.E., Oliver M.J. (2017). Water deficit-induced changes in transcription factor expression in maize seedlings. Plant Cell Environ..

[42] Delauney A.J., Verma D.P.S. (1993). Proline biosynthesis and osmoregulation in plants. Plant J..

[43] Sharp R.E., Davies W.J. (1979). Solute regulation and growth by roots and shoots of water-stressed maize plants. Planta.

[44] Westgate M.E., Boyer J.S. (1985). Osmotic adjustment and the inhibition of leaf, root, stem and silk growth at low water potentials in maize. Planta.

[45] Sharp R.E., Hsiao T.C., Silk W.K. (1990). Growth of the maize primary root at low water potentials. II. Role of growth and deposition of hexose and potassium in osmotic adjustment. Plant Physiol..

[46] Voetberg G.S., Sharp R.E. (1991). Growth of the maize primary root at low water potentials. III. Role of increased proline deposition in osmotic adjustment. Plant Physiol..

[47] Zhang L., Peng J., Chen T.T., Zhao X.H., Zhang S.P., Liu S.D., Dong H.L., Feng L., Yu S.X. (2014). Effect of drought stress on lipid peroxidation and proline content in cotton roots. J. Anim. Plant Sci..

[48] Ahmad N., Malagoli M., Wirtz M., Hell R. (2016). Drought stress in maize causes differential acclimation responses of glutathione and sulfur metabolism in leaves and roots. BMC Plant Biol..

[49] Ober E.S., Sharp R.E. (1994). Proline accumulation in maize (*Zea mays* L.) primary roots at low water potentials. I. Requirement for increased levels of abscisic acid. Plant Physiol..

[50] Ashraf M., Foolad M.R. (2007). Roles of glycine betaine and proline in improving plant abiotic stress resistance. Environ. Exp. Bot..

[51] Lum M.S., Hanafi M.M., Rafii Y.M., Akmar A.S.N. (2014). Effect of drought stress on growth, proline and antioxidant enzyme activities of upland rice. J. Anim. Plant Sci..

[52] Moulin M., Deleu C., Larher F., Bouchereau A. (2006). The lysine-ketoglutarate reductase–saccharopine dehydrogenase is involved in the osmo-induced synthesis of pipecolic acid in rapeseed leaf tissues. Plant Physiol. Biochem..

[53] Nishizawa A., Yabuta Y., Shigeoka S. (2008). Galactinol and raffinose constitute a novel function to protect plants from oxidative damage. Plant Physiol..

[54] Van den Ende W. (2013). Multifunctional fructans and raffinose family oligosaccharides. Front. Plant Sci..

[55] van der Rest B., Boisson A.M., Gout E., Bligny R., Douce R. (2002). Glycerophosphocholine metabolism in higher plant cells. Evidence of a new glyceryl-phosphodiester phosphodiesterase. Plant Physiol..

[56] Larsson K.E., Nyström B., Liljenberg C. (2006). A phosphatidylserine decarboxylase activity in root cells of oat (*Avena sativa*) is involved in altering membrane phospholipid composition during drought stress acclimation. Plant Physiol. Biochem..

[57] Cheng L., Bucciarelli B., Liu J., Zinn K., Miller S., Patton-Vogt J., Allan D., Shen J., Vance C.P. (2011). White lupin cluster root acclimation to phosphorus deficiency and root hair development involve unique glycerophosphodiester phosphodiesterases. Plant Physiol..

[58] Du Y., Zhao Q., Chen L., Yao X., Zhang W., Zhang B., Xie F. (2020). Effect of drought stress on sugar metabolism in leaves and roots of soybean seedlings. Plant Physiol. Biochem..

[59] Verslues P.E., Sharma S. (2010). Proline metabolism and its implications for plant-environment interaction. Arab. Book.

[60] Kopriva S., Malagoli M., Takahashi H. (2019). Sulfur nutrition: Impacts on plant development, metabolism, and stress responses. J. Exp. Bot..

[61] Morgan P.W., Drew M.C. (1997). Ethylene and plant responses to stress. Physiol. Plant..

[62] Sobeih W.Y., Dodd I.C., Bacon M.A., Grierson D., Davies W.J. (2004). Long-distance signals regulating stomatal conductance and leaf growth in tomato (*Lycopersicon esculentum*) plants subjected to partial root-zone drying. J. Exp. Bot..

[63] Haworth I.S., Rodger A., Richards W.G. (1991). A molecular mechanics study of spermine complexation to DNA: A new model for spermine-poly (dG-dC) binding. Proc. R. Soc. Lond. Ser. B Biol. Sci..

[64] Chan K.X., Wirtz M., Phua S.Y., Estavillo G.M., Pogson B.J. (2013). Balancing metabolites in drought: The sulfur assimilation conundrum. Trends Plant Sci..

[65] Sweetlove L.J., Beard K.F.M., Nunes-Nesi A., Fernie A.R., Ratcliffe R.G. (2010). Not just a circle: Flux modes in the plant TCA cycle. Trends Plant Sci..

[66] Bouché N., Fromm H. (2004). GABA in plants: Just a metabolite?. Trends Plant Sci..

[67] Liu C., Zhao L., Yu G. (2011). The dominant glutamic acid metabolic flux to produce γ-amino butyric acid over proline in *Nicotiana tabacum* leaves under water stress relates to its significant role in antioxidant activity. J. Integr. Plant Biol..

[68] Gakière B., Hao J., de Bont L., Pétriacq P., Nunes-Nesi A., Fernie A.R. (2018). NAD+ biosynthesis and signaling in plants. Crit. Rev. Plant Sci..

[69] Wei M., Zhuang Y., Li H., Li P., Huo H., Shu D., Huang W., Wang S. (2020). The cloning and characterization of hypersensitive to salt stress mutant, affected in quinolinate synthase, highlights the involvement of NAD in stress-induced accumulation of ABA and proline. Plant J..

[70] Noctor G., Foyer C.H. (1998). Ascorbate and glutathione: Keeping active oxygen under control. Annu. Rev. Plant Physiol. Plant Mol. Biol..

[71] Noctor G., Foyer C.H. (2016). Intracellular redox compartmentation and ROS-related communication in regulation and signaling. Plant Physiol..

[72] Nayyar H., Gupta D. (2006). Differential sensitivity of C3 and C4 plants to water deficit stress: Association with oxidative stress and antioxidants. Environ. Exp. Bot..

[73] Tausz M., Šircelj H., Grill D. (2004). The glutathione system as a stress marker in plant ecophysiology: Is a stress-response concept valid?. J. Exp. Bot..

[74] Foyer C.H., Noctor G. (2011). Ascorbate and glutathione: The heart of the redox hub. Plant Physiol..

[75] Vernoux T., Wilson R.C., Seeley K.A., Reichheld J.P., Muroy S., Brown S., Maughan S.C., Cobbett C.S., Van Montagu M., Inzé D. (2000). The root meristemless1/cadmium sensitive2 gene defines a glutathione-dependent pathway involved in initiation and maintenance of cell division during postembryonic root development. Plant Cell.

[76] Diaz Vivancos P., Dong Y., Ziegler K., Markovic J., Pallardó F.V., Pellny T.K., Verrier P.J., Foyer C.H. (2010). Recruitment of glutathione into the nucleus during cell proliferation adjusts whole-cell redox homeostasis in *Arabidopsis thaliana* and lowers the oxidative defence shield. Plant J..

[77] Voothuluru P., Mäkelä P., Zhu J., Yamaguchi M., Cho I.-J., Oliver M.J., Simmonds J., Sharp R.E. (2020). Apoplastic hydrogen peroxide in the growth zone of the maize primary root. Increased levels differentially modulate root elongation under well-watered and water-stressed conditions. Front. Plant Sci..

[78] Lappartient A.G., Touraine B. (1997). Glutathione-mediated regulation of ATP sulfurylase activity, SO_4_^2−^ uptake, and oxidative stress response in intact canola roots. Plant Physiol..

[79] Munné-Bosch S. (2005). The role of α-tocopherol in plant stress tolerance. J. Plant Physiol..

[80] Zhang H., Ni Z., Chen Q., Guo Z., Gao W., Su X., Qu Y. (2016). Proteomic responses of drought-tolerant and drought-sensitive cotton varieties to drought stress. Mol. Genet. Genom..

