# Supplementary material for: Antioxidant Metabolism Underlies Different Metabolic Strategies for Primary Root Growth Maintenance under Water Stress in Cotton and Maize
Source: Antioxidants (Basel). 2022 Apr 22;11(5):820. doi: 10.3390/antiox11050820 (PMC9137980; doi:10.3390/antiox11050820)
Supplement: Supplementary file 1 [file antioxidants-11-00820-s001.zip › Figure S1.pdf]

Supplementary Materials

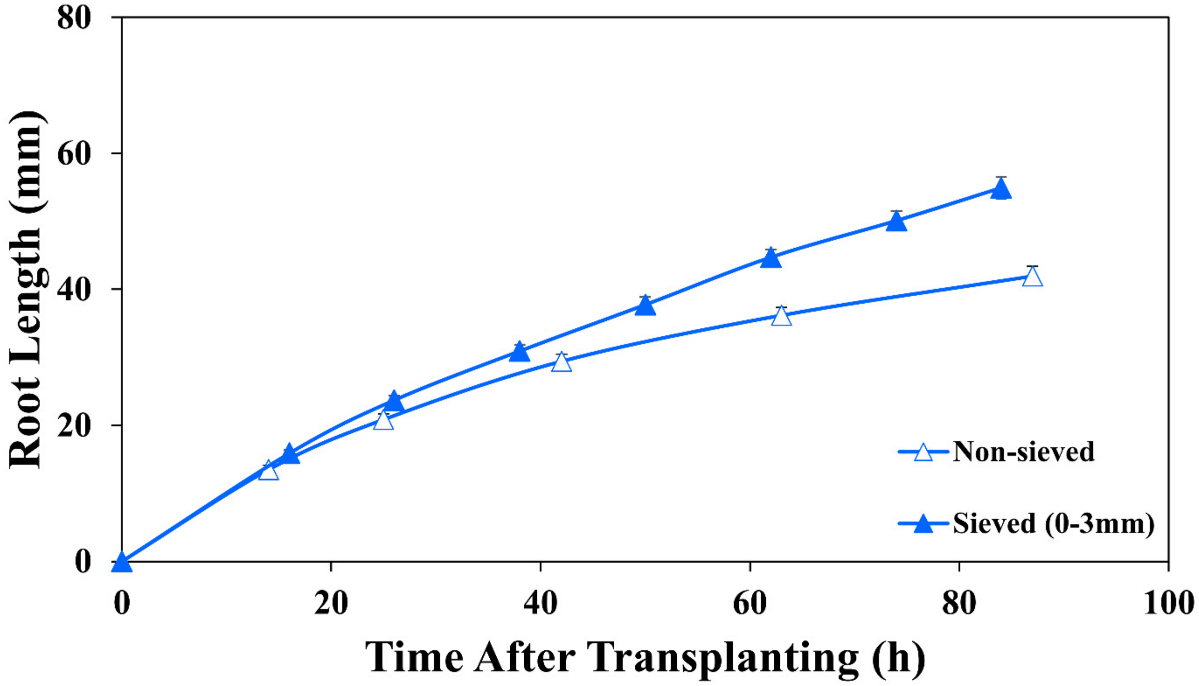

Figure S1. Time course of primary root length for cotton (cv. AU90810) seedlings after transplanting to vermiculite at a water potential of −1.6 MPa. The vermiculite was either non-sieved or sieved to a particle size of 3 mm or less to enhance the hydraulic contact between the roots and the vermiculite particles. Data are means ± SE (n = 29–38).
