# Supplementary material for: Antioxidant Metabolism Underlies Different Metabolic Strategies for Primary Root Growth Maintenance under Water Stress in Cotton and Maize
Source: Antioxidants (Basel). 2022 Apr 22;11(5):820. doi: 10.3390/antiox11050820 (PMC9137980; doi:10.3390/antiox11050820)
Supplement: Supplementary file 1 [file antioxidants-11-00820-s001.zip › Table S1.pdf]

Supplementary Materials

Table S1. Root growth characteristics of cotton and maize primary roots under well-watered and water-stressed conditions

	Root Elongation Rate (mm h^{-1})			Final Cell Length (µm)			Cell Flux (cells h^{-1})		
	WW	WS (−1.0 MPa)	WS (−1.6 MPa)	WW	WS (−1.0 MPa)	WS (−1.6 MPa)	WW	WS (−1.0 MPa)	WS (−1.6 MPa)
**Cotton**	2.10 ± 0.02	1.04 ± 0.03	0.54 ± 0.03	158 ± 3	135 ± 4	101 ± 3	13.2 ± 0.3	7.6 ± 0.1	5.4 ± 0.2

	WS/WW Root Elongation Rate		WS/WW Final Cell Length		WS/WW Cell Flux	
	−1.0 MPa	−1.6 MPa	−1.0 MPa	−1.6 MPa	−1.0 MPa	−1.6 MPa
**Cotton**	49.5%	25.7%	85.4%	63.9%	57.6%	40.9%

	Root Elongation Rate (mm h^{-1})		Final Cell Length (µm)		Cell Flux (cells h^{-1})	
	WW	WS (−1.6 MPa)	WW	WS (−1.6 MPa)	WW	WS (−1.6 MPa)
**Maize**	2.57 ± 0.02	1.00 ± 0.00	190 ± 3	142 ± 4	13.6 ± 0.3	7.1 ± 0.2

	WS/WW Root Elongation Rate	WS/WW Final Cell Length	WS/WW Cell Flux
**Maize**	38.9%	74.7%	52.2%

Elongation rate, final cortical cell length, and cell flux in well-watered (WW) and water-stressed (WS) primary roots of cotton (cv. AU90810) and maize (cv. FR697), and the ratios of the traits (expressed as percentages) between water-stressed and well-watered conditions. In the water stress treatments, vermiculite water potentials were −1.0 MPa (cotton) and −1.6 MPa (cotton and maize). Data are means ± SE of 5–6 roots, and were collected at 48 h (cotton) or 36 h (maize) after transplanting when root elongation was steady in all treatments (Figure 1). The maize data are reproduced from Voothuluru et al. [77].
