# Supplementary material for: Antioxidant Metabolism Underlies Different Metabolic Strategies for Primary Root Growth Maintenance under Water Stress in Cotton and Maize
Source: Antioxidants (Basel). 2022 Apr 22;11(5):820. doi: 10.3390/antiox11050820 (PMC9137980; doi:10.3390/antiox11050820)
Supplement: Supplementary file 1 [file antioxidants-11-00820-s001.zip › Table S2.pdf]

Table S2. Ratios of metabolite abundance in R1 of the elongation zone between water-stressed and well-watered primary roots of cotton and maize.

Sub Pathway	Metabolite	Cotton	Maize
		Region 1	Region 1
Amines and poly-amines	5-methylthioadenosine (MTA)	1.56	NS
	nicotianamine	0.51	0.56
	putrescine	0.37	NS
	N-acetylputrescine	NS	0.72
Aromatic amino acid metabolism (PEP derived)	3-(4-hydroxyphenyl)lactate	4.06	NS
	tryptamine	4.97	1.97
	tyramine	3.41	6.89
	tyrosine	1.35	NS
	phenylalanine	0.72	NS
	3-methoxytyrosine	0.58	NS
	quinate	0.51	NS
	kynurenate	0.48	NS
	tryptophan	NS	1.55
	phenylpyruvate	NS	0.39
	N-methylphenylalanine		26.44
	O-methyl tyrosine		0.55
	phenyllactate (PLA)		0.23
Aspartate family (OAA derived)	2-hydroxyadipate	2.43	NS
	N-methylpipecolate	3.92	3.44
	N-acetyl-β-alanine	3.09	0.65
	methionine	3.08	NS
	S-adenosylmethionine (SAM)	2.59	NS
	lysine	2.29	NS
	homoserine lactone	2.07	NS
	methionine sulfoxide	1.93	0.3
	alanine	1.84	NS
	N-acetylalanine	0.72	NS
	aspartate	0.48	0.74
	S-adenosylhomocysteine (SAH)	0.43	NS
	asparagine	NS	3.87
	6-oxopiperidine-2-carboxylic acid	NS	2.11
	allo-threonine	NS	1.83
	N6-carboxymethyllysine	NS	1.75
	threonine	NS	1.53
	S-methylmethionine	NS	0.8
	N-acetylaspartate (NAA)	NS	0.21
	N-acetylasparagine	NS	0.11

	N-formylmethionine	NS	0.5
	N-acetylthreonine		2.07
	N6-carboxyethyllysine		1.64
	2-piperidinone		1.19
	N-6-trimethyllysine		0.7
**Branched Chain Amino Acids (OAA derived)**	allo-isoleucine		0.56
**BCAA-isoleucine catabolism**	2-methylglutarate	2.58	1.7
**Branched Chain Amino Acids (pyruvate derived)**	methylsuccinate	5.31	1.47
	leucine	1.82	NS
	α-hydroxyisovalerate	1.78	2.34
	2,3-dihydroxyisovalerate	0.58	NS
	2-isopropylmalate	0.46	NS
	3-methyl-2-oxobutyrate	NS	6.85
	3-hydroxyisobutyrate	NS	0.56
	4-methyl-2-oxopentanoate	NS	0.4
	N-methylleucine		6.95
	3-hydroxy-2-ethylpropionate		1.55
	levulinate (4-oxovalerate)		0.54
	norvaline		0.5
**Glutamate family (alpha-ketoglu-tarate derived)**	pyroglutamine	12.16	NS
	stachydrine	6.68	NS
	N-acetylproline	4.65	1.75
	1-methylhistidine	4.54	NS
	trans-4-hydroxyproline	3.89	3.47
	3-methylhistidine	1.94	NS
	carboxyethyl-GABA	1.92	0.41
	homocitrulline	1.64	0.67
	N-δ-acetylornithine	0.82	NS
	glutamate	0.72	0.71
	N-acetylglutamate	0.64	0.49
	N-monomethylarginine	0.6	NS
	4-guanidinobutanoate	0.72	NS
	N-acetylarginine	0.58	NS
	N-methylglutamate	NS	1.64
	arginine	NS	1.47
	ornithine	NS	0.67
	dimethylarginine (SDMA + ADMA)	NS	0.52
	N-acetylglutamine	NS	0.28
	4-acetamidobutanoate	NS	0.58
	1,3-diaminopropane		0.53

	4-hydroxy-2-oxoglutaric acid		0.17
Glutathione metabolism	ophthalmate	4.42	NS
	γ-glutamylmethionine	3.83	NS
	γ-glutamylleucine	2.5	NS
	glutathione, reduced (GSH)	0.71	41.38
	glutathione, oxidized (GSSG)	0.68	6.4
	cysteine-glutathione disulfide	0.58	NS
	γ-glutamylglutamate	0.53	NS
	γ-glutamyltryptophan	NS	2.52
	γ-glutamylglycine	NS	1.67
	γ-glutamylthreonine	NS	1.87
	γ-glutamylglutamine	NS	0.47
	4-hydroxy-nonenal-glutathione		11.72
	norophthalmate		1.64
Serine family (phosphoglycer-ate derived)	betaine	4.2	NS
	serine	1.76	2.58
	N-acetylserine	0.55	0.53
	N-acetyltaurine	0.82	2.15
	cysteine	NS	1.7
	sulfate	NS	1.22
	O-acetylserine		0.53
	homocysteine		0.39
Amino sugar and nucleotide sugar	arabonate	1.65	NS
	UDP-*N*-acetylglucosamine	0.67	0.7
	ribose	0.62	NS
	UDP-*N*-acetylgalactosamine	0.68	NS
	N-acetylglucosamine 6-phosphate	0.67	3.05
	ribulose	NS	0.48
	ribonate	NS	0.41
	xylulose		0.62
	maltol		0.5
	xylitol		0.74
C5 branched dibasic acid metabolism	itaconate (methylenesuccinate)		0.46
Calvin cycle and pentose phosphate	ribose 5-phosphate		0.61
	ribose 1-phosphate		0.52
Glycolysis	pyruvate	1.94	0.53
	lactate	1.92	NS
	glucuronate	0.6	NS

	glucose 6-phosphate	**0.59**	**NS**
**Inositol metabolism**	inositol 1-phosphate (I1P)	**0.41**	**NS**
**Photorespiration**	oxalate (ethanedioate)	**0.84**	**NS**
**Sucrose, glucose, fructose metabolism**	galactinol	**5.08**	
	galactonate	**1.61**	**NS**
	mannose-6-phosphate	**0.67**	**NS**
	mannitol	**0.73**	**NS**
	3-deoxyoctulosonate	**0.73**	**NS**
	maltotriose	**NS**	**1.43**
	galactose 1-phosphate	**NS**	**0.64**
	methyl glucopyranoside (α + β)	**NS**	**0.55**
	1-kestose		**3.78**
	sucrose-6-phosphate		**2.34**
	erythrose		**2.16**
	glucoheptose		**0.29**
**TCA cycle**	citrate	**0.43**	**0.38**
	aconitate	**0.31**	**0.63**
	isocitrate	**0.24**	**NS**
	succinate	**0.75**	**0.5**
	α-ketoglutarate	**NS**	**3.57**
	malate	**NS**	**0.67**
	maleate	**NS**	**0.41**
**Carnitine metabolism**	deoxycarnitine	**11.05**	**NS**
**Ascorbate metabolism**	gulonate	**NS**	**0.64**
**CoA metabolism**	phosphopantetheine		**4.59**
**Nicotinate and nicotinamide metabolism**	trigonelline (N'-methylnicotinate)	**2.32**	**0.39**
	nicotinate ribonucleoside	**0.75**	**0.43**
	nicotinamide adenine dinucleotide (NAD+)	**0.62**	**0.76**
	nicotinate	**0.42**	**NS**
	nicotinamide ribonucleotide (NMN)	**NS**	**0.67**
	nicotinamide	**NS**	**0.35**
	nicotinamide riboside	**NS**	**0.27**
**Oxidative phosphorylation**	methylphosphate	**0.68**	**NS**

Riboflavin and FAD metabolism	flavin adenine dinucleotide (FAD)	0.51	0.35
	flavin mononucleotide (FMN)	0.4	NS
Thiamine metabolism	thiamin (Vitamin B1)	0.52	NS
Vitamin B metabolism (B6 or B12)	pyridoxine (Vitamin B6)	0.67	NS
	pyridoxal	0.55	0.59
Choline metabolism	2-dimethylaminoethanol		0.1
Fatty acid, Amino	2-aminoheptanoate	3.45	NS
Fatty acid, Dicarboxylate	pimelate (heptanedioate)	7	0.61
	glutarate (pentanedioate)	3.06	NS
	malonate	2.03	1.66
	adipate	1.48	0.65
	azelate (nonanedioate)	0.81	NS
	suberate (octanedioate)	0.71	NS
	ethylmalonate		0.46
	dodecanedioate		0.33
Free fatty acid	3-hydroxybutyrate (BHBA)	0.79	NS
	linolenate [α or γ; (18:3n3 or 6)]	0.41	NS
	2-hydroxypalmitate	NS	0.57
	arachidate (20:0)	NS	0.53
	2-hydroxystearate	NS	0.5
	eicosenoate (20:1)	NS	0.33
	palmitate (16:0)	NS	0.75
	oleate (18:1n9)		0.57
	heptanoate (7:0)		0.33
Phospholipids	choline	0.8	NS
	1-palmitoyl-GPE (16:0)	0.71	NS
	1-linoleoyl-GPE (18:2)	0.69	NS
	1-oleoyl-GPE (18:1)	0.6	0.57
	ethanolamine	0.56	NS
	1-palmitoyl-GPA (16:0)	0.54	NS
	1-oleoyl-GPA (18:1)	0.47	0.32
	1-palmitoyl-GPG (16:0)	0.62	NS
	1-oleoyl-GPG (18:1)	0.48	0.34
	1-linoleoyl-GPI (18:2)	0.44	0.26
	glycerophosphoethanolamine	NS	31.92
	glycerophosphoglycerol	NS	27.39
	glycerophosphoinositol	NS	6.62

	glycerol 3-phosphate	NS	6.5
	1-palmitoyl-2-oleoyl-GPC (16:0/18:1)	NS	2.59
	1-palmitoyl-2-linoleoyl-GPC (16:0/18:2)	NS	2.39
	glycerol	NS	1.25
	1-oleoyl-2-linoleoyl-GPI (18:1/18:2)	NS	0.47
	1-palmitoyl-2-oleoyl-GPE (16:0/18:1)	NS	0.36
	1-oleoyl-GPI (18:1)	NS	0.21
	1-palmitoylglycerol (16:0)	NS	0.43
	1-palmitoyl-2-arachidonoyl-GPC (16:0/20:4)		3.81
	2-palmitoylglycerol (16:0)		0.41
	1-oleoylglycerol (18:1)		0.45
**Sphingolipid**	sphingosine	0.52	0.11
	sphinganine	NS	0.18
**Sterols**	stigmasterol	1.68	0.44
	campesterol	NS	0.57
	3-hydroxy-3-methylglutarate	NS	0.57
	fucosterol	NS	0.5
**Purine metabolism**	adenosine 3'-monophosphate (3'-AMP)	9.39	0.36
	guanosine 3'-monophosphate (3'-GMP)	3.94	NS
	adenosine-2',3'-cyclic monophosphate	2.78	NS
	inosine 5'-monophosphate (IMP)	2.32	0.6
	adenosine 2'-monophosphate (2'-AMP)	1.55	NS
	N6-carbamoylthreonyladenosine	0.76	NS
	adenosine 5'-monophosphate (AMP)	0.61	NS
	guanosine 5'- monophosphate (5'-GMP)	0.49	1.51
	N6-succinyladenosine	0.49	NS
	guanosine	0.47	0.62
	N2-methylguanosine	0.44	NS
	N1-methyladenosine	0.35	NS
	N6-methyladenosine	0.3	NS
	xanthine	0.24	NS
	adenine	0.24	NS
	2'-deoxyinosine	0.21	NS
	2'-deoxyguanosine	0.12	0.61
	2'-deoxyadenosine	0.11	0.64
	2'-deoxyadenosine 5'-monophosphate	0.11	NS
	2'-deoxyguanosine 5'-monophosphate (dGMP)	0.11	NS
	guanine	0.67	NS
	7-methylguanosine	0.58	NS
	N1-methylguanosine	0.49	NS
	allantoin	NS	3.04
	N2,N2-dimethylguanosine	NS	2.94

	allantoic acid	NS	0.5
	inosine	NS	0.27
	2'-O-methyladenosine	NS	0.63
	urate	NS	0.27
	2'-deoxyadenosine 3'-monophosphate		0.45
	2'-deoxyguanosine 3'-monophosphate		0.45
Pyrimidine metabolism	β-alanine	3.03	3.6
	uridine 2'-monophosphate (2'-UMP)	0.61	NS
	cytidine 3'-monophosphate (3'-CMP)	0.59	NS
	uridine	0.53	0.56
	orotate	0.52	0.22
	uracil	0.47	NS
	pseudouridine	0.41	NS
	cytidine 5'-monophosphate (5'-CMP)	0.32	0.56
	5-methylcytidine	0.25	NS
	5-methyluridine (ribothymidine)	0.2	NS
	5-methyl-2'-deoxycytidine	0.18	0.46
	thymidine	0.17	0.66
	thymidine 5'-monophosphate	0.15	NS
	2'-deoxycytidine 5'-monophosphate	0.14	0.63
	2'-deoxycytidine	0.1	0.42
	3-ureidopropionate	0.65	NS
	cytidine	NS	0.51
	cytosine	NS	0.11
	3-aminoisobutyrate		1.86
	thymidine 3'-monophosphate		0.36
	ectoine		0.07
Dipeptide	cys-gly, oxidized	2.54	4.85
	glutaminylleucine	1.28	NS
	leucylglycine	1.65	NS
	alanylleucine	NS	0.7
	threonylphenylalanine	NS	0.68
	phenylalanylalanine	NS	0.46
	leucylglutamine	NS	0.61
	leucylphenylalanine/isoleucylphenylalanine		0.36
Benzenoids	hydroquinone β-D-glucopyranoside	0.62	NS
Fatty acid and sugar derivatives	galactarate (mucic acid)	NS	0.6
Phenylpropanoids	ferulate	1.76	NS
	4-hydroxycinnamate	0.53	2.15

	vanillate	**NS**	**1.85**
	3,4-dimethoxycinnamic acid		**4.74**
	coniferyl aldehyde		**1.4**
**Terpenoids**	mevalonate		**0.53**
	mevalonolactone		**0.29**
**Chemicals**	succinimide		**0.2**

In the water stress treatments, vermiculite water potentials were −1.0 MPa (cotton) and −1.6 MPa (maize), which resulted in equivalent root tip water potentials in the two species (Figure 2). The fold changes are the averages of comparisons of the water-stressed treatment to well-watered developmental and temporal controls. Yellow and blue cells indicate significant increases or decreases in abundance, respectively (darker shades, $p < 0.05$ in all comparisons; lighter shades, $0.05 < p < 0.10$ in at least one comparison). White cells indicate non-significant (NS) changes in at least one comparison. Gray cells indicate that the metabolite was not detected. No minimum cutoff value was applied to the fold changes.
