# Supplementary material for: Antioxidant Metabolism Underlies Different Metabolic Strategies for Primary Root Growth Maintenance under Water Stress in Cotton and Maize
Source: Antioxidants (Basel). 2022 Apr 22;11(5):820. doi: 10.3390/antiox11050820 (PMC9137980; doi:10.3390/antiox11050820)
Supplement: Supplementary file 1 [file antioxidants-11-00820-s001.zip › Table S3.pdf]

Table S3. Ratios of metabolite abundance in R2 of the elongation zone between water-stressed and well-watered primary roots of cotton and maize.

Sub Pathway	Metabolite	Cotton Region 2	Maize Region 2
Amines and polyamines	5-methylthioadenosine (MTA)	0.57 / 0.63	NS
	N-acetylputrescine	0.55 / 0.4	NS
	agmatine	0.17 / 0.11	NS
	spermidine	0.09 / 0.07	NS
	putrescine	0.03 / 0.02	NS
	nicotianamine	NS	0.36 / 0.56
Aromatic amino acid metabolism (PEP derived)	3-(4-hydroxyphenyl)lactate	6.42 / 4.16	0.51 / 0.44
	tryptamine	3.89 / 1.95	NS
	tyramine	3.36 / 1.85	NS
	tryptophan	1.81 / 1.34	NS
	quinate	0.49 / 0.54	2.02 / 2.31
	phenylalanine	0.47 / 0.37	0.78 / 0.53
	phenylpyruvate	0.29 / 0.28	0.17 / 0.29
	N-acetylphenylalanine	0.51 / 0.55	0.30 / 0.38
	tyrosine	NS	0.62 / 0.38
	4-hydroxyphenylpyruvate	NS	0.25 / 0.17
	N-methylphenylalanine		10.73 / 7.04
	O-methyl tyrosine		0.58 / 0.66
Aspartate family (OAA derived)	*N*-methylpipecolate	4.62 / 4.72	NS
	2-hydroxyadipate	2.22 / 2.63	NS
	N-acetyl-β-alanine	2.17 / 2.65	NS
	lysine	1.85 / 1.25	NS
	6-oxopiperidine-2-carboxylate	2.7 / 1.77	2.72 / 2.6
	alanine	0.77 / 0.45	NS
	N-acetylasparagine	0.54 / 0.63	0.16 / 0.24
	N-acetylmethionine	0.53 / 0.56	NS
	N-formylmethionine	0.5 / 0.7	0.11 / 0.17
	S-adenosylmethionine (SAM)	0.47 / 0.46	1.57 / 1.65
	S-adenosylhomocysteine (SAH)	0.29 / 0.45	NS
	aspartate	0.54 / 0.7	NS
	asparagine	NS	2.41 / 1.77
	homoserine	NS	0.73 / 0.63
	homoserine lactone	NS	0.7 / 0.41
	methionine	NS	0.37 / 0.26
	N-acetylaspartate (NAA)	NS	0.32 / 0.43
	methionine sulfoxide	NS	0.28 / 0.23
	2-piperidinone		1.23 / 1.22

Branched Chain Amino Acids (pyruvate derived)	methylsuccinate	**2.15 / 1.62**	**NS**
	α-hydroxyisovalerate	**1.56 / 2.32**	**NS**
	3-methyl-2-oxobutyrate	**0.52 / 0.8**	**1.98 / 2.05**
	3-methyl-2-oxovalerate	**0.48 / 0.78**	**NS**
	4-methyl-2-oxopentanoate	**0.48 / 0.69**	**NS**
	2-isopropylmalate	**0.3 / 0.4**	**NS**
	2,3-dihydroxyisovalerate	**0.19 / 0.18**	**0.32 / 0.24**
	leucine	**NS**	**0.57 / 0.39**
	N-acetylleucine	**NS**	**0.36 / 0.45**
	N-methylleucine		**4.62 / 3.4**
	norvaline		**0.47 / 0.57**
BCAA-isoleucine catabolism	2-methylglutarate	**1.65 / 1.74**	**NS**
Glutamate family (α-ketoglutarate derived)	1-methylhistidine	**4.6 / 3.76**	**NS**
	pyroglutamine	**7.8 / 6.65**	**NS**
	N-acetylproline	**3.97 / 6.12**	**NS**
	stachydrine	**3.53 / 3.57**	**NS**
	homocitrulline	**2.44 / 2.49**	**NS**
	3-methylhistidine	**1.72 / 1.82**	**NS**
	carboxyethyl-GABA	**1.51 / 1.77**	**NS**
	N-monomethylarginine	**0.58 / 0.43**	**NS**
	arginine	**0.56 / 0.38**	**NS**
	N-acetylarginine	**0.4 / 0.51**	**NS**
	N-acetylglutamine	**0.32 / 0.39**	**0.17 / 0.45**
	trans-4-hydroxyproline	**NS**	**2.21 / 2.26**
	N-methylglutamate	**NS**	**1.43 / 1.6**
	dimethylarginine (SDMA + ADMA)	**NS**	**0.53 / 0.58**
	2-pyrrolidinone		**4.73 / 8.51**
	1,3-diaminopropane		**0.35 / 0.49**
	4-hydroxy-2-oxoglutaric acid		**0.27 / 0.61**
Glutathione metabolism	γ-glutamyltyrosine	**1.95 / 3.48**	**NS**
	γ-glutamyltryptophan	**1.91 / 2.44**	**4.43 / 5.53**
	γ-glutamylleucine	**1.32 / 1.53**	**NS**
	cysteine-glutathione disulfide	**0.51 / 0.35**	**NS**
	glutathione, oxidized (GSSG)	**0.49 / 0.47**	**NS**
	glutathione, reduced (GSH)	**0.38 / 0.49**	**NS**
	γ-glutamylglutamine	**0.2 / 0.41**	**NS**
	γ-glutamylalanine	**NS**	**2.98 / 3.85**
	γ-glutamylthreonine	**NS**	**2.22 / 3.28**
	γ-glutamylphenylalanine	**NS**	**1.46 / 1.86**
	betaine	**2.85 / 2.59**	**NS**

**Serine family (phospho-glycerate derived)**	sulfate	**0.65 / 0.44**	**1.22 / 1.14**
	glycine	**0.5 / 0.3**	**NS**
	N-acetylserine	**0.35 / 0.45**	**NS**
	N-acetyltaurine	**NS**	**2.32 / 2.61**
	serine	**NS**	**2.12 / 1.52**
	homocysteine		**0.16 / 0.13**
	O-acetylserine		**0.14 / 0.16**
**Amino sugar and nucleotide sugar**	arabonate	**1.44 / 1.4**	**NS**
	xylose	**0.72 / 0.55**	**NS**
	ribose	**0.53 / 0.51**	**NS**
	UDP-galactose	**0.47 / 0.23**	**NS**
	UDP-*N*-acetylglucosamine	**0.37 / 0.67**	**NS**
	UDP-*N*-acetylgalactosamine	**0.55 / 0.71**	**NS**
	ribonate	**NS**	**0.44 / 0.49**
	xylitol		**0.63 / 0.65**
	xylulose		**0.51 / 0.65**
	ribulose/xylulose 5-phosphate		**0.31 / 0.45**
	maltol		**0.25 / 0.59**
**C5 branched dibasic acid metabolism**	itaconate (methylenesuccinate)		**0.35 / 0.42**
**Calvin cycle and pentose phosphate**	ribose 5-phosphate		**0.34 / 0.54**
	ribose 1-phosphate		**0.27 / 0.41**
**Glycolysis**	glucuronate	**0.53 / 0.5**	**0.71 / 0.62**
	pyruvate	**NS**	**0.5 / 0.7**
	fructose-6-phosphate	**NS**	**0.22 / 0.2**
**Photorespiration**	oxalate (ethanedioate)	**0.78 / 0.77**	**NS**
**Sucrose, glucose, fructose metabolism**	galactinol	**17.49 / 18.17**	
	methyl glucopyranoside (α + β)	**2.64 / 2.41**	**NS**
	3-deoxyoctulosonate	**0.52 / 0.5**	**NS**
	maltose	**0.48 / 0.57**	**NS**
	mannose-6-phosphate	**NS**	**0.44 / 0.47**
	1-kestose		**5.55 / 5.37**
	sucrose-6-phosphate		**1.92 / 1.92**
**TCA cycle**	succinate	**0.6 / 0.52**	**NS**
	fumarate	**0.6 / 0.52**	**NS**
	malate	**0.52 / 0.48**	**NS**
	maleate	**0.34 / 0.39**	**NS**
	α-ketoglutarate	**NS**	**2.27 / 2.89**
	2-methylcitrate	**NS**	**5.02 / 4.43**

	aconitate	NS	0.74 / 0.84
	citrate	NS	0.4 / 0.53
	mesaconate (methylfumarate)		0.73 / 0.69
**Ascorbate metabolism**	gulonate	NS	0.48 / 0.47
**Carnitine metabolism**	deoxycarnitine	7.37 / 5.38	NS
**Nicotinate and nicotinamide metabolism**	trigonelline (N'-methylnicotinate)	2.99 / 2.65	NS
	nicotinate ribonucleoside	0.65 / 0.65	NS
	nicotinate	0.55 / 0.44	1.85 / 2.33
	nicotinamide adenine dinucleotide (NAD+)	0.39 / 0.48	NS
	nicotinamide riboside	NS	0.43 / 0.35
**Oxidative phosphorylation**	acetylphosphate	NS	0.4 / 0.37
**Riboflavin and FAD metabolism**	flavin adenine dinucleotide (FAD)	0.43 / 0.55	NS
	flavin mononucleotide (FMN)	0.49 / 0.57	NS
**Thiamine metabolism**	thiamin (Vitamin B1)	0.61 / 0.52	2.17 / 2.41
**Tocopherol metabolism**	α-tocopherol	0.5 / 0.52	NS
**Vitamin B metabolism (B6 or B12)**	pyridoxate	2.51 / 1.88	NS
	pyridoxal	0.37 / 0.46	NS
**Auxin metabolism**	indoleacetate	0.59 / 0.41	NS
	2-oxindole-3-acetate	NS	0.58 / 0.55
**Fatty acid, Amino**	2-aminoheptanoate	3.32 / 2.85	NS
**Fatty acid, Dicarboxylate**	pimelate (heptanedioate)	4.60 / 5.19	NS
	malonate	1.98 / 1.56	NS
	azelate (nonanedioate)	0.53 / 0.76	2.24 / 2.44
	adipate	NS	0.6 / 0.79
	dodecanedioate		0.2 / 0.33
**Free fatty acid**	2-hydroxyglutarate	0.52 / 0.52	NS
	3-hydroxybutyrate (BHBA)	0.37 / 0.49	NS
	linolenate [α or γ; (18:3n3 or 6)]	0.11 / 0.16	NS
	linoleate (18:2n6)	0.1 / 0.14	NS
	oleate (18:1n9)		0.45 / 0.71
**Phospholipids**	glycerophosphoethanolamine	1.67 / 2.08	15.74 / 8.8
	glycerophosphoinositol	1.35 / 2.36	3.77 / 3.92

	choline	0.73 / 0.77	NS
	1-palmitoyl-GPA (16:0)	0.42 / 0.47	NS
	ethanolamine	0.42 / 0.45	NS
	1-oleoyl-GPA (18:1)	0.25 / 0.33	NS
	1-palmitoyl-2-oleoyl-GPE (16:0/18:1)	0.25 / 0.46	NS
	1-oleoyl-GPE (18:1)	0.21 / 0.38	NS
	1-oleoyl-GPC (18:1)	0.18 / 0.42	NS
	1-palmitoyl-GPI (16:0)	0.39 / 0.49	2.61 / 3
	1-palmitoyl-2-linoleoyl-GPE (16:0/18:2)	0.38 / 0.56	NS
	1-palmitoyl-GPG (16:0)	0.25 / 0.51	1.80 / 4.05
	glycerophosphoglycerol	NS	8.08 / 6.62
	glycerol 3-phosphate	NS	2.16 / 2.45
**Sphingolipid**	phytosphingosine	0.43 / 0.54	NS
	sphingosine	0.24 / 0.46	NS
	sphinganine	0.22 / 0.38	0.1 / 0.13
**Sterols**	3-hydroxy-3-methylglutarate	NS	0.52 / 0.58
	stigmasterol	NS	0.45 / 0.52
	campesterol	NS	0.5 / 0.65
	fucosterol	NS	0.3 / 0.38
	ergosterol		1.29 / 1.29
**Purine metabolism**	adenosine 3'-monophosphate (3'-AMP)	9.08 / 6.86	NS
	guanosine 3'-monophosphate (3'-GMP)	6.57 / 2.48	0.15 / 0.07
	adenosine-2',3'-cyclic monophosphate	2.90 / 3.28	0.52 / 0.24
	allantoin	0.66 / 0.53	1.88 / 1.17
	N2,N2-dimethylguanosine	0.6 / 0.56	1.73 / 4
	7-methylguanosine	0.56 / 0.36	NS
	inosine	0.44 / 0.4	0.19 / 0.58
	adenosine 5'-monophosphate (AMP)	0.43 / 0.57	NS
	allantoic acid	0.42 / 0.32	0.17 / 0.2
	guanosine 5'- monophosphate (5'-GMP)	0.38 / 0.51	NS
	guanosine	0.32 / 0.42	NS
	adenine	0.26 / 0.29	NS
	inosine 5'-monophosphate (IMP)	0.23 / 0.49	NS
	2'-deoxyguanosine	0.20 / 0.48	NS
	2'-deoxyadenosine	0.18 / 0.35	NS
	2'-deoxyadenosine 5'-monophosphate	0.11 / 0.37	NS
	hypoxanthine	0.6 / 0.39	NS
	adenosine 2'-monophosphate (2'-AMP)	NS	0.55 / 0.51
	guanosine-2',3'-cyclic monophosphate	NS	0.55 / 0.27
	xanthine	NS	0.28 / 0.25
	urate	NS	0.41 / 0.35
	guanosine 2'-monophosphate (2'-GMP)		0.75 / 0.72

Pyrimidine metabolism	cytidine 2',3'-cyclic monophosphate	**2.90 / 2.06**	**0.67 / 0.44**
	uridine-2',3'-cyclic monophosphate	**1.91 / 2.02**	**0.59 / 0.39**
	uridine 5'-monophosphate (UMP)	**0.47 / 0.58**	**NS**
	5-methylcytidine	**0.37 / 0.32**	**NS**
	uridine	**0.36 / 0.47**	**NS**
	thymidine	**0.27 / 0.35**	**NS**
	thymidine 5'-monophosphate	**0.23 / 0.51**	**NS**
	cytidine 5'-monophosphate (5'-CMP)	**0.23 / 0.33**	**NS**
	uracil	**0.2 / 0.35**	**NS**
	2'-deoxycytidine 5'-monophosphate	**0.19 / 0.46**	**NS**
	uridine 3'-monophosphate (3'-UMP)	**NS**	**0.7 / 0.76**
	cytosine	**NS**	**0.14 / 0.24**
	cytidine 3'-monophosphate (3'-CMP)	**NS**	**0.8 / 0.71**
	cytidine	**NS**	**0.71 / 0.77**
	uridine 2'-monophosphate (2'-UMP)	**NS**	**0.61 / 0.69**
	ectoine		**0.13 / 0.2**
Dipeptide	alanylleucine	**0.75 / 0.66**	**NS**
	valylglycine	**0.74 / 0.69**	**NS**
	glycylleucine	**0.61 / 0.71**	**NS**
	cys-gly, oxidized	**NS**	**6.57 / 7.58**
	phenylalanylalanine	**NS**	**0.5 / 0.62**
Benzenoids	hydroquinone β-D-glucopyranoside	**0.42 / 0.36**	**NS**
Fatty acid and sugar derivatives	galactarate (mucic acid)	**NS**	**0.6 / 0.81**
Phenylpropanoids	4-hydroxycinnamate	**0.31 / 0.44**	**NS**
	vanillate	**0.34 / 0.5**	**2.26 / 2.27**
	3,4-dimethoxycinnamic acid		**11.88 / 10.84**
Terpenoids	mevalonate		**0.38 / 0.69**
	mevalonolactone		**0.22 / 0.49**
Chemicals	succinimide		**0.31 / 0.43**

In the water stress treatments, vermiculite water potentials were −1.0 MPa (cotton) and −1.6 MPa (maize), which resulted in equivalent root tip water potentials in the two species (Figure 2). The fold changes are the averages of comparisons of the water-stressed treatment to well-watered developmental and temporal controls. Comparisons of water-stressed R2 (WSR2) to both well-watered R2 (WWR2) and R3 (WWR3) are shown in each cell as (WSR2/WWR2) / (WSR2/WWR3). Yellow and blue cells indicate significant increases or decreases in abundance, respectively (darker shades, $p < 0.05$ in all comparisons; lighter shades, $0.05 < p < 0.10$ in at least one comparison). White cells indicate non-significant (NS) changes in at least one comparison. Gray cells indicate that the metabolite was not detected. No minimum cutoff value was applied to the fold changes.
